# The first dipeptidyl peptidase III from a thermophile: Structural basis for thermal stability and reduced activity

**DOI:** 10.1371/journal.pone.0192488

**Published:** 2018-02-08

**Authors:** Igor Sabljić, Marko Tomin, Mihaela Matovina, Iva Sučec, Ana Tomašić Paić, Antonija Tomić, Marija Abramić, Sanja Tomić

**Affiliations:** 1 Division of Physical Chemistry, Ruđer Bošković Institute, Zagreb, Croatia; 2 Division of Organic Chemistry and Biochemistry, Ruđer Bošković Institute, Zagreb, Croatia; Russian Academy of Medical Sciences, RUSSIAN FEDERATION

## Abstract

Dipeptidyl peptidase III (DPP III) isolated from the thermophilic bacteria *Caldithrix abyssi* (*Ca*) is a two-domain zinc exopeptidase, a member of the M49 family. Like other DPPs III, it cleaves dipeptides from the N-terminus of its substrates but differently from human, yeast and *Bacteroides thetaiotaomicron* (mesophile) orthologs, it has the pentapeptide zinc binding motif (HEISH) in the active site instead of the hexapeptide (HEXXGH). The aim of our study was to investigate structure, dynamics and activity of *Ca*DPP III, as well as to find possible differences with already characterized DPPs III from mesophiles, especially *B*. *thetaiotaomicron*. The enzyme structure was determined by X-ray diffraction, while stability and flexibility were investigated using MD simulations. Using molecular modeling approach we determined the way of ligands binding into the enzyme active site and identified the possible reasons for the decreased substrate specificity compared to other DPPs III. The obtained results gave us possible explanation for higher stability, as well as higher temperature optimum of *Ca*DPP III. The structural features explaining its altered substrate specificity are also given. The possible structural and catalytic significance of the HEISH motive, unique to *Ca*DPP III, was studied computationally, comparing the results of long MD simulations of the wild type enzyme with those obtained for the HEISGH mutant. This study presents the first structural and biochemical characterization of DPP III from a thermophile.

## Introduction

*Caldithrix abyssi* is a thermophilic and anaerobic Gram-negative bacteria isolated from a Mid-Atlantic Ridge hydrothermal vent [1]. It is the first cultivated representative of a phylum-level bacterial lineage. The genome of *Caldithrix abyssi* was sequenced within the framework of Genomic Encyclopedia of Bacteria and Archaea (GEBA) project [2, 3]. The genomic analysis revealed the presence of more than 150 genes encoding peptidases, both extracellular and intracellular, in agreement with the ability of *C*. *abyssi* to grow on complex proteinaceous substrates, such as beef extract, soy bean, peptone and yeast extract [4]. None of the *C*. *abyssi* peptidases have been characterized functionally or biochemically.

During our investigation of the metallopeptidase family M49 diversity (MEROPS [5], merops.sanger.ac.uk) we have found that a 558 amino acid protein from *C*. *abyssi* DSM 13497 (UniProtKB entry: H1XW48) represents an ortholog of this family, also known as the dipeptidyl peptidase III (DPP III) family. DPP III (EC 3.4.14.4) is a cytosolic zinc-metallopeptidase which cleaves dipeptides from the N-termini of its substrates, consisting of three to ten amino acids [6, 7]. It is broadly distributed in eukaryotic cells and considered to participate in normal intracellular protein catabolism. Mammalian enzyme is involved in cellular defense against oxidative stress, as an activator of the Keap1-Nrf2 signaling pathway [8, 9]. DPP III was thought to be an exclusively eukaryotic enzyme until 2003, when first two bacterial genome sequences of (*Bacteroides thetaiotaomicron* and *Porphyromonas gingivalis*) that contain *DPP3* gene appeared in the UniProt KB [10]. The deduced amino acid sequences of two prokaryotic orthologs showed low homology with eukaryotic ones. However, they enabled the recognition of five evolutionary conserved amino acid sequence regions in the M49 family, including the unique hexapeptide zinc-binding motif HEXXGH. Biochemical properties of the eukaryotic DPPs III have been studied extensively [7], and the crystal structures of two representatives, human and yeast have been solved [11, 12]. Differently, the only prokaryotic DPP III characterized so far is the recombinant DPP III from *Bacteroides thetaiotaomicron* [13].

The DPP III family is rapidly growing due to the newly sequenced genomes which enable comparative studies and novel discoveries. Recently, we have determined the diversity in M49 family with regard to the protein sequence length through bioinformatic analyses [14, 15]. We also found that the conserved hexapeptide HEXXGH signature motif is reduced to pentapeptide HEXXH in some of the new members. Since it was assumed that hexapeptide zinc binding motif (HEXXGH), established as a “hallmark” of the DPP III family, is required for the hydrolytic activity, these findings pose the question of the M49 peptidases active site “core” as well as of the enzymatic properties of the members with pentapeptide signature motif. Among the members containing the pentapeptide signature motif, we have noted two large subgroups. One consisting of the plant proteins, about 750–800 amino acids long, which besides the five conserved regions of M49 family, contain the so called NUDIX motif, a characteristic of Nudix hydrolases [16], at the N-terminal part of the sequence. The other subgroup comprises numerous shorter bacterial homologues with the sequence length of ~ 550 amino acids, none of them being functionally characterized. Among them, we have chosen the uncharacterized protein from *C*. *abyssi* DSM 13497 (UniProt: H1XW48) as a subject of our research for several reasons: it represents the subgroup of bacterial homologs with pentapeptide signature motif, it is much smaller than other M49 peptidases (675 to 738 amino acids) characterized up to date, and, according to XtalPred server [17], it is classified in optimal crystallization class proteins.

In this work we present biochemical characterization of *C*. *abyssi* DPP III, its crystal structure and the results of the molecular modeling, docking combined with molecular dynamics (MD) simulations, hydrogen bond analysis and the relative binding free energy calculations. This study should help to resolve the question of the enzymatic “core”, i.e. the minimal number of the conserved residues in the enzyme active site required for catalytic activity of a M49 family member. Namely, we were interested in whether the HEXXH pentapeptide is sufficient for the peptidase activity. Further on, the study was also aimed at investigating possible structural characteristics responsible for different temperature optimum and thermal inactivation of the thermophilic *vs* mesophilic enzymes from M49 family.

## Materials and methods

### Cloning

The *Cabys_2252* gene encoding DPP III enzyme was amplified from the genomic DNA of *Caldithrix abyssi* using PCR with forward 5’-CCGGCTCGAGATTGCTATTTCCC-3’ and reverse 5’-GGGCCGGCATATGATGAAACGAA-3’ primers, respectively. The PCR product was then cloned into *Nde*I and *Xho*I restriction sites of the pET-21a(+) vector. Resulting construct contained hexa-histidine tag (-LEHHHHHH) at the C terminal end of the enzyme and a spontaneous mutation K21R. This construct was used solely for crystallization purposes. *C*. *abyssi DPP3* gene was amplified from the pET-21a(+) vector, cloned in the pLATE31 vector by using aLICator Ligation Independent Cloning and Expression System Protein kit (Thermo Scientific, USA), and the spontaneous mutation K21R was corrected. Protein expressed from the pLATE31 vector was used for the biochemical characterization of *C*. *abyssi* DPP III.

### Overexpression, purification, and characterization of *Ca*DPP III proteins

Heterologous expression of the DPP III protein was done using *Escherichia coli* BL21-CodonPlus(DE3)-RIL cells transformed with previously described constructs. Further procedure was performed as described for the human DPP III (h.DPP) [18], with the exceptions that the induction was done using 0.25 mg/mL of IPTG and the culture was grown at 18 °C overnight. Bacterial cells were harvested by centrifugation at 5600 g at 4 °C for 10 min and stored at -20 °C until purification. Selenomethionine (Se-Met) labeled DPP III was produced by transforming *Escherichia coli* B834(DE3) cells with the previously described construct. Overexpression of Se-Met labeled DPP III was accomplished according to the procedure used for the DPP III from *Bacteroides thetaiotaomicron* [19], with the exception that induction was done using 0.25 mg/mL of IPTG.

The samples for purification were prepared according to the protocol described by I. Sabljic *et al* [19]. The purification was performed in two steps: affinity chromatography on Ni-NTA resin (5 mL prepacked His-trap FF, GE Healthcare) and gel filtration on column with Superdex 200 (GE Healthcare) previously equilibrated with 50 mM Tris-HCl (pH 7.4) containing 100 mM NaCl. Both steps were done with ÄKTA FPLC (GE Healthcare). The purity of the protein was analyzed by SDS-PAGE, on 12% gel plate, and the protein concentration was determined by measurement of *A*_280_ using theoretical molar extinction coefficient, 49865 M^-1^ cm^-1^. For the long-term storage, the enzyme was kept at –80 °C.

### Enzyme activity assay and kinetic analysis

Temperature and pH optimum for the *Ca*DPP III enzymatic activity were determined by the standard assay at temperatures from 25 to 70 ° with Arg_2_-2-naphthylamide (Arg_2_-2NA) as the substrate, using the colorimetric method, as previously described [20, 21].

PH optimum was determined at pH 6 to 7 in 50 mM sodium-phosphate buffer, and at pH 7 to 8.6 in 50 mM Tris HCl buffer at 37 °C and 50 °C, with the addition of 50 μM CoCl_2_, by the standard assay with Arg_2_-2NA as the substrate [20].

In our earlier studies of the DPP III orthologs, we assumed that thermal stability of an enzyme is closely related to its activity and have used activity tests with Arg_2_-2NA to determine an enzyme thermal stability. In line with this assumption and our previous studies on DPP III enzymes the thermal stability of *Ca*DPP III was determined by heating the reaction mixture without the substrate for 30 min on temperatures between 37 and 80 °C, and, after cooling on ice for 2 min, performing the standard activity test with Arg_2_-2NA substrate at 37 °C, as previously described [18].

Substrate specificity and the influence of inhibitors and effectors on enzymatic activity were measured at 50 °C and at pH 7.0, with the addition of 50 μM CoCl_2_, and substrate concentration was 40 μM.

Kinetic parameters for hydrolysis of Arg_2_-2NA and Gly-Arg-2NA were determined at pH 7.0, in the presence of 50 μM CoCl_2_. For the complex with Arg_2_-2NA the measurements were performed at 25 °C and 50 °C, and for the complex with Gly-Arg-2NA only at 50 °C. The initial rate measurements were carried out on Cary Eclipse Fluorescence Spectrophotometer (Agilent) using excitation and emission wavelength of 332 nm and 420 nm, respectively. The enzyme was preincubated for 2 min at either 25 °C or 50 °C, and the reaction started with the addition of substrate. The initial rate was determined from the continuous measurement (duration of 1 min) of fluorescence of the free 2-naphthylamine product. The kinetic parameters were calculated using nonlinear regression analysis of three kinetic measurements in *GraphPad Prism* software (GraphPad Software, Inc., USA), with Arg_2_-2NA concentrations from 5 to 300 μM and Gly-Arg concentrations from 15 to 350 μM.

### Crystallization and data collection

For crystallization experiments the protein sample was concentrated up to 10.2 mg/mL. Crystallization screening was done by vapor diffusion method in sitting drops using Orxy8 robot (Douglas Instruments, UK). The drops were prepared by mixing 0.5 μL of protein solution and 0.5 μL of crystallization solution at 20 °C. Two commercial screens were used: Morpheus from Molecular Dimensions (Newmarket, UK) and Index from Hampton Research (California, USA). Crystals were obtained in three conditions: Index D7 (0.1 M Bis-Tris pH 6.5, 25% PEG 3,350), Index D9 (0.1 M Tris pH 8.5, 25% PEG 3,350) and Index D10 (0.1 M Bis-Tris pH 6.5, 20% PEG 5,000). Further optimization was done by hanging drop technique in 24 well Linbro plates. Best diffracting crystals where grown by using 900 μL of 200 mM (NH_4_)_2_SO_4_ as reservoir solution and by mixing 1 μL of the protein solution with 1 μL of the original Index D7 crystallization solution.

To crystallize the Se-Met labeled DPP III protein samples were concentrated up to 12.6 mg/mL. Crystallization was performed by sitting drop technique in 24 well Linbro plates. Best diffracting crystals were obtained using 600 μL of the homemade Index D10 crystallization solution as reservoir solution while the drops were made of 1 μL of the protein solution and 1 μL of the original Index D10 crystallization solution.

Prior to flash-cooling in liquid N_2_, all crystals were cryoprotected by soaking for a few seconds in the original Index D7 or D10 crystallization solutions supplemented with 20% ethylene glycol. Diffraction experiments were carried out at 100 K at Elettra Sincrotrone Trieste (Trieste, Italy) with a PILATUS 2M detector. Optimal energies for MAD experiment were obtained by scanning the x-ray energy against the fluorescence emitted by the sample. For MAD experiments two datasets were collected from a single crystal at wavelengths 0.9793 Å (peak) and 0.9796 Å (inflection). The crystal diffracted up to resolution of 2.8 Å. Dataset for unlabeled protein was collected at 1.2705 Å up to 2.1 Å resolution. Data collection and refinement statistics are summarized in Table 1.

**Table 1:** Data collection, structure determination, and refinement statistics^a^.

	*Ca*DPP III
**Crystal parameters**	
Resolution (Å)	2.1
Space group	*P*2_1_2_1_2_1_
Unit cell parameters	
*a*, *b*, *c* (Å)	51.47, 90.55, 133.0
*α*, *β*, *γ* (°)	90, 90, 90
Matthews coefficient (Å^3^/Da)	2.37
Solvent content (%)	48
**Data collection**	
Completeness (%)	99.8 (98.4)
No. of unique reflections	36965
*I*/σ(*I*)	13.4 (2.4)
R_merge_	0.117 (0.819)
CC_1/2_	0.998 (0.903)
Redundancy	8.7 (8.3)
Wilson B-factor (Å^2^)	29.4
**Refinement**	
*R* / *R*_free_	0.210 / 0.240
RMSD bonds length (Å)	0.003
Average *B* factor	38.3
Number of molecules in a.u.	1
No. of atoms	
Protein	4254
Ligand	15
Zinc ion	1
Water molecules	335
Ramachandran analysis^b^	
Favored (%)/*n*	98.1
Allowed (%)/*n*	1.9
Outlier (%)/*n*	0

^a^ Data for the highest resolution shell are given in parentheses.

The abbreviations: RMSD stands for Root Mean Square Deviation and a.u. for asymmetric unit.

^b^ Defined by the validation program MOLPROBITY [34].

### Phasing, model building, and refinement

All collected datasets were processed using XDS [22] and data scaling was done using Aimless [23]. For calculation of the *R*_free_, 5% of reflections was randomly selected and excluded from all refinements.

The initial model was obtained using the dataset collected for the Se-Met labeled protein using MAD method from AutoSol [24] within PHENIX software suite [25]. Using this model, the partial structure of unlabeled protein was determined by molecular replacement method (MOLREP [26]). Further improvement of the electron density maps was done using the program *Parrot* [27]. The last step of automated model building was done using the BUCCANEER [28] program. Although this procedure improved the quality of the initial model, the complete structure was not obtained. Using programs COOT [29], REFMAC [30, 31] and PHENIX [25], alternately, the final structure was obtained. The COOT program was used for model building, fitting and the real space refinement against *σ*_A_-weighted 2*F*_o_-*F*_c_ and *F*_o_-*F*_c_ electron density maps, while REFMAC and PHENIX were used for refinement. Translation, rotation, and screw-rotation (TLS) parameterization of anisotropic displacement was used in the last refinement step [32]. The final model is missing 34 amino acid residues; 31 at the N- and 3 at the C-terminus. Data collection and refinement statistics are given in Table 1. Final coordinates and structure factors have been deposited in the Protein Data Bank (accession number 6EOM). Programs Aimless, MOLREP, Parrot, BUCCANEER and REFMAC are part of the CCP4 software suite [33].

### Computational methods

#### System parametrization and preparation

The experimentally determined structure of the ligand-free *Ca*DPP III was used as the initial structure for the molecular modeling study. The amino acids residues missing at the N-terminus of the experimentally determined structure, Met1 –Lys31, were partially (Cys19 –Lys31) reconstructed using the program Modeller 9.14 [35], while the amino acid residues 1–18 were omitted since we assumed that this is a signaling peptide based on the SignalP [36] server prediction (S1 Fig).

All Arg and Lys residues in our model are positively charged (+1e) while Glu and Asp residues are negatively charged (-1e), as expected at physiological conditions. The protonation of histidines was checked according to their ability to form hydrogen bonds with neighboring amino acid residues or to coordinate the metal ion. The HEISH to HEISGH mutation was performed using Modeller 9.14 [35]. The protein parametrization was performed within the ff14SB force field [37], while the substrate was parametrized using the generalized amber force field (gaff2). The missing parameters were obtained through the Antechamber module [38]. For the zinc cation, Zn^2+^, parameters derived in previous work were used [39]. All calculations were performed using the Amber16 suite of programs [40]. The proteins and protein-substrate complexes were placed into the truncated octahedron box filled with TIP3P water molecules [41] and Na^+^ ions [42] were added in order to neutralize the systems. Additionally, a single chlorine ion bound to the protein surface in the vicinity of Thr231 and Ile232 was kept throughout the simulations.

#### MD simulations

Prior to molecular dynamics simulations, the protein geometry was optimized in three cycles with different constraints. In the first cycle (1500 steps), the protein and zinc atoms were restrained by the harmonic potential with a force constant of 32 kcal mol^-1^ Å^-1^. In the second (2500 steps) cycle, the same force was applied to the protein backbone while the zinc atom was relaxed. In the third cycle (1500 steps), no constraints were applied. During the first period of equilibration (30 ps of gentle heating from 0 to 300K), the *NVT* ensemble was used, while the second period of equilibration (170 ps at 300 K), as well as all of the following simulations were performed at constant temperature and pressure (300K and 1 atm, the *NpT* ensemble) using a time step of 1 fs. The equilibrated structure was subjected either to 200 ns of *NpT* conventional MD (cMD) or 200 ns of accelerated MD (aMD) simulations using the 2 fs time step. The temperature was held constant using Langevin dynamics [43] with a collision frequency of 1 ps^−1^. Pressure was regulated by a Berendsen barostat [44]. Bonds involving hydrogen atoms were constrained using the SHAKE [45, 46] algorithm.

The aMD simulations were performed using double boost potential. Besides the torsional, the total potential energy term was also boosted enhancing the diffusion of the explicit solvent molecules around the biomolecule. The average total potential energy, $\overset{-}{E_{pot}}$, and the average torsional potential energy, $\overset{-}{E_{dih}}$, for the simulated systems, were obtained from the first 50 ns of cMD simulations (S1 Table). The values of *E*_*r*_ and *E*_*a*_ parameters (1 and 0.1 kcal mol^-1^, energy per residue and atom, respectively) used to calculate the potential energy boost *E*_T_(*r*), the torsional potential energy boost *E*_t_(*r*), and the parameters controlling the boost potentials roughness, *α*_T_ and *α*_t_ have been taken from our previous works [47].

The substrates (Arg_2_-2NA, Gly-Arg-2NA, Gly-Phe-2NA and Gly-Pro-2NA) were docked in the equilibrated enzyme structure in order to mimic binding determined for the human DPP III complexes.^45^ Complex structures were optimized using the same procedure as for the ligand-free enzyme and were heated over the course of 50 ns. We performed 100 ns of cMD followed by 50 ns of aMD simulations (S2 Table) for the each of the *Ca*DPP III—substrate complexes using the same conditions as for the unligated enzyme.

HEISGH mutant was simulated for 200 ns using cMD, while the simulations of its complex with Arg_2_-2NA were conducted in a same way as the wild-type complex.

#### MM-PBSA

The substrate binding free energies were approximated by the MM-PBSA energies using the AMBER16 [40] implementation. For each complex the MM-PBSA energies have been calculated on a set of 5 ns long intervals sampled throughout the trajectory. The calculations were performed using a salt concentration of 0.15 M. The MM-GBSA calculations, utilizing GB model of Onufriev et al. [48], have been performed as well. Internal and external dielectric constants for MM-PBSA calculations were set at their default values of 1.0 and 80.0, respectively.

#### Data analysis

**Fig 1:**
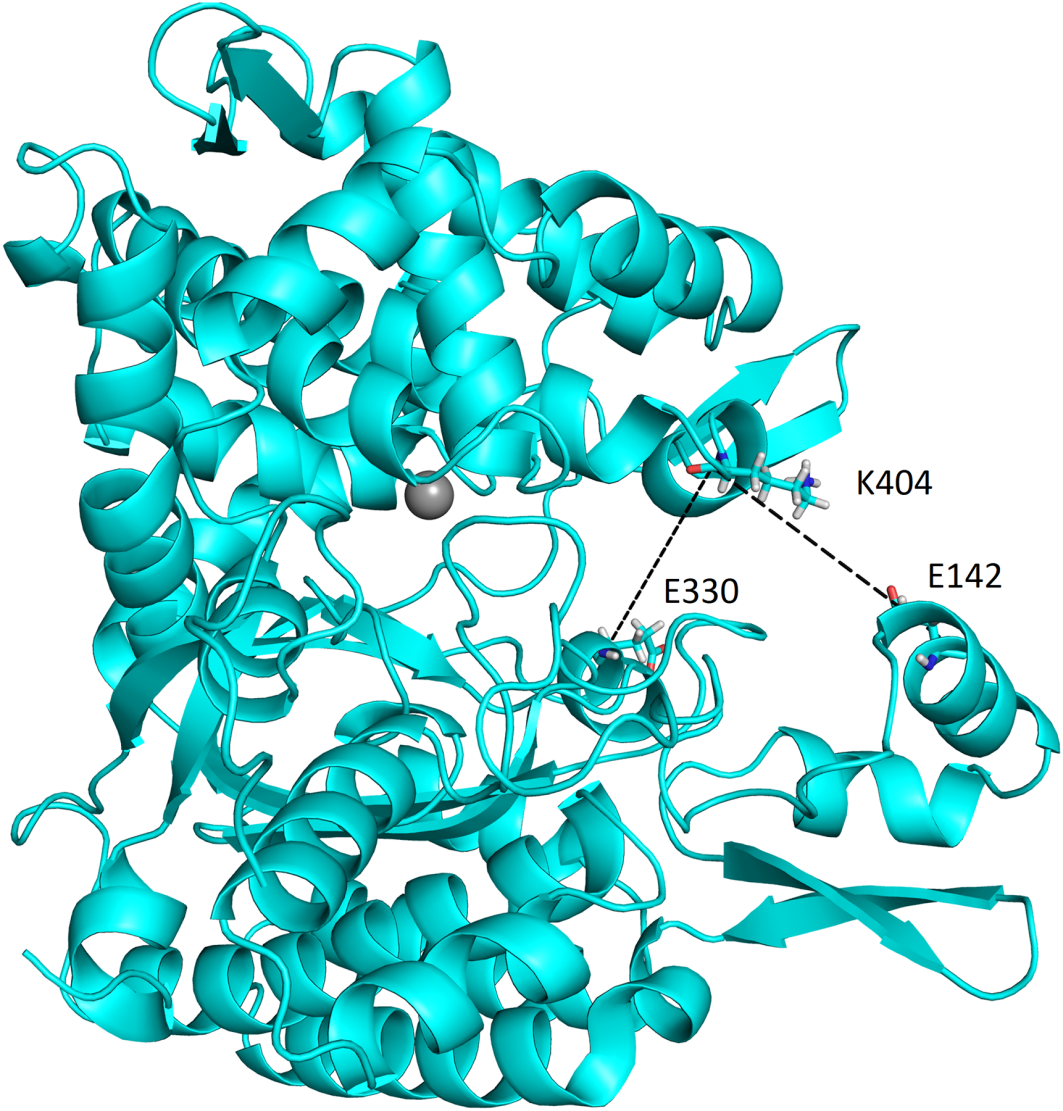
Interactions between Arg_2_-2NA and the amino acid residues in the binding site of the HEISGH *Ca*DPP III mutant. The figure was prepared using the PyMol program [51].

In order to analyze and to characterize the conformational space of *Ca*DPP III, as well as to determine the most prominent protein motions, several types of data analysis were performed. All calculations were performed with CPPTRAJ module of the AmberTools program package [49]. Based on previous work on bacterial DPP III [50], besides the radius of gyration, we traced 2 additional geometric parameters (Fig 1) during the simulations.

Intermolecular hydrogen bond analysis was performed on the same trajectory sections as MM-PBSA calculations in order to closely examine the ligand-protein interactions. For the hydrogen bond definition default distance and angle cut-off values were used (3.0 Å and 135°, respectively). These relatively tight criteria ensured that only the most relevant interactions were taken into account. The hydrogen bonds population is calculated as the ratio of the number of trajectory frames the hydrogen bond is present in and the total number of frames (H_bpop_ = N(frames with Hbond)/N(frames total)). In the case of residue forming multiple hydrogen bonds, a sum of these values is given, which allows values larger than 100%. For example if a glutamate forms hydrogen bonds with both carboxyl oxygens at the same time, the sum of hydrogen bonds might be above 100%. Such approach enabled better quantification of an amino acid residue importance in substrate stabilization while keeping the table dimensions within reasonable boundaries.

## Results and discussion

### Biochemical properties of *Ca*DPP III

Temperature optimum of *Ca*DPP III was determined by measuring enzymatic activity towards Arg_2_-2NA at temperatures from 25 °C to 70 °C. Enzyme showed the maximum activity at 50 °C. pH optimum was determined at 37 °C and at 50 °C in the pH range from 6 to 8.6. The activity between pH 6 and 7 was determined in 50 mM sodium-phosphate buffer, and between 7 and 8.6 in 50 mM Tris-HCl buffer (S3 Table). The maximum activity at both temperatures was determined at pH 7.0, however the activity of *Ca*DPP III at pH 7.0 in phosphate buffer was only 58% of the activity in Tris-HCl buffer at the same pH at 37 °C, and 20% of the activity in Tris-HCl buffer at 50 °C, therefore, activities measured in phosphate buffer are not good comparison to the Tris-HCl buffer. However, since Tris-HCl cannot be used to prepare buffers of pH lower than 7, we used this approximation.

Thermal stability was determined at temperatures from 37 °C to 80 °C. The reaction mixture without Arg_2_-2NA substrate was held at the appropriate temperature for 30 min, after which it was transferred to ice for 2 min, and the residual activity towards Arg_2_-2NA was determined by the standard activity test at 37 °C. The highest residual activity determined at 50 °C was 72.8 nmol min^-1^ mg^-1^. The activity dropped below 50% at 70 °C (S2 Fig). As expected, *Ca*DPP III exhibits higher thermal stability than h.DPP III, and *Bt*DPP III, which are almost completely inactivated at 55 °C and 50 °C, respectively [13, 18, 19].

Substrate specificity was determined at 50 °C and pH 7.0. *Ca*DPP III showed the highest activity towards Gly-Arg-2NA substrate (Table 2).

**Table 2:** Relative activity of *Ca*DPP III towards different (di)peptidyl-2-naphthylamides.

Substrate	RA / %
Arg-Arg-2NA	100
Ala-Arg-2NA	65.1
Gly-Arg-2NA	167.1
Phe-Arg-2NA	129.5
Pro-Arg-2NA	143.5
Ala-Ala-2NA	103.2
Gly-Phe-2NA	8.7
Gly-Pro-2NA	1.6
His-Phe -2NA	37.5
His-Ser-2NA	26.5
Lys-Ala-2NA	68.4
Glu-His-2NA	14.2
Arg-2NA	2.6
BANA	0.3

RA—relative activity in comparison with Arg_2_-2NA substrate (specific activity towards Arg_2_-2NA was 97 nmol min^-1^ mg^-1^); BANA—N-benzoyl-d,l-arginine-β-naphthylamide

Unlike all other DPPs III characterized so far which show high substrate specificity towards Arg_2_-2NA [13, 18], *Ca*DPP III has similar substrate specificity towards five different dipeptidyl-2-NA substrates, Arg_2_-2NA, Gly-Arg-2NA, Pro-Arg-2NA, Phe-Arg-2NA and Ala-Ala-2NA.

We tested the influence of several effectors on the *Ca*DPP III activity towards Arg_2_-2NA and Gly-Arg-2NA. The tests were performed at 50 °C and pH 7 (S4 Table). The metal chelators EDTA and O-phenantroline abolished the enzyme activity towards both substrates, as expected, while other agents, known to have an effect on h.DPP III, did not have a substantial influence on the *Ca*DPP III activity towards Arg_2_-2NA. However, the sulfhydryl agents iodoacetamide (IAM) and 4,4’-Dithiodipyridine (DTDP) lowered the activity towards Gly-Arg-2NA to around 27% and 43%, respectively. Interestingly GSH increased *Ca*DPP III activity by 50%. Similar effect of GSH was noticed earlier in the case of yDPP III. The incubation of yDPP III with 0.1 mM GSH resulted in 2.3 folds higher activity [52]. One possible explanation is that GSH increases the activity of DPP III enzymes by reducing reactive cysteines. Since yDPP III has 4 cysteins while *Ca*DPP III has only one the effect GSH has on the enzyme activity is more pronounced for yDPP III than for *Ca*DPP III. Addition of metal ions does not have significant effect on the enzyme specific activity (S5 Table).

The kinetic parameters were determined for both Arg_2_-2NA, which is the preferred substrate for most DPPs III characterized so far, and Gly-Arg-2NA towards which *Ca*DPP III showed around 60% higher specific activity than towards Arg_2_-2NA (Table 2). The results of the kinetic measurements showed that, despite lower specific activity, the kinetic efficiency of hydrolysis (*k*_*ca*t_/*K*_*M*_) is 11 times higher for the Arg_2_-2NA due to almost 10 times higher *K*_*M*_ value for Gly-Arg-2NA, which makes Arg_2_-2NA a better substrate after all. Kinetic parameters for the hydrolysis of Arg_2_-2NA at 25 °C were also measured (Table 3). *K*_*M*_ for the hydrolysis was 10 times higher than in the case of human and *Bt*DPP III, while *k*_*ca*t_ is an order of magnitude lower than *Bt*DPP III, and 2 order of magnitudes lower than h.DPP III. Consequently, the efficiency of hydrolysis is 3 orders of magnitude lower than for human and *Bt*DPP III [13, 18, 53].

**Table 3:** Kinetic parameters for the hydrolysis of Arg_2_-2NA and Gly-Arg-2NA substrates by *Ca*DPP III.

Substrate	*K*_*M*_ / μM	*k*_cat_ /s^-1^	*k*_cat_/*K*_*M*_ / 10^6^ M^-1^ s^-1^
Arg_2_-2NA	35.2 ± 2.0	3.07 ± 0.04	0.08730
Gly-Arg-2NA	328.9 ± 36.5	2.53 ± 0.17	0.00767
Arg_2_-2NA at 25 °C*	30.5 ± 3.9	0.20 ± 0.04	0.00663

The kinetic parameters were determined from the initial reaction rates at 50 °C in 50 mM Tris-HCl buffer pH 7.0, as described in Materials and Methods section. *K*_*M*_ and *k*_*cat*_ are the averages of three measurements ± SD.

*The kinetic parameters for Arg_2_-2NA determined at 25 °C are the averages of three measurements ± SD

### Crystal structure of *Ca*DPP III

**Fig 2:**
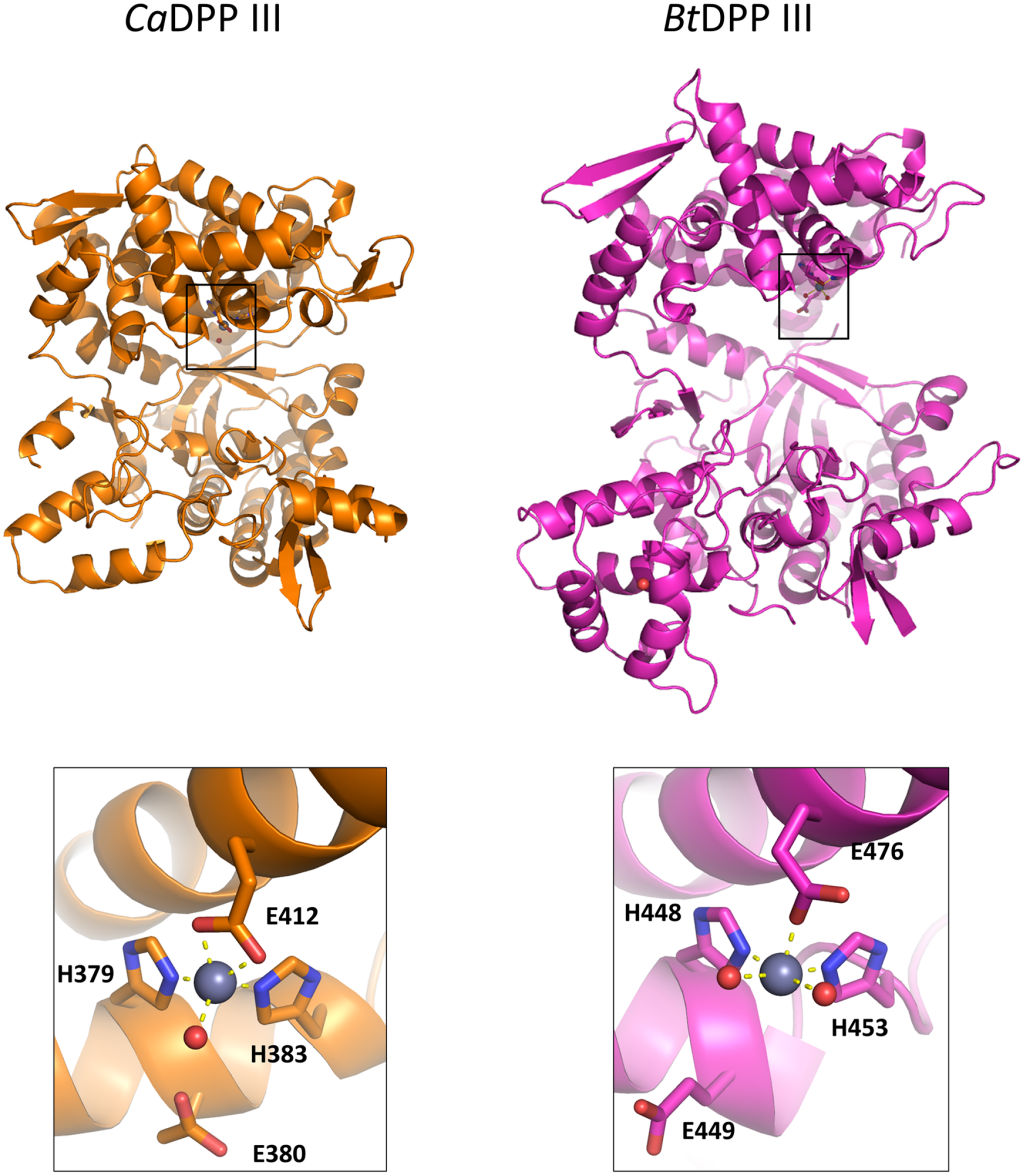
3D structures of DPP III from *Caldithrix abyssi* and *Bacteroides thetaiotaomicron* (PDB ID: 5NA7) determined by X-ray diffraction (up) and close-up views of their zinc binding sites (below). Amino acids coordinating the zinc ions (shown as grey spheres).

*Ca*DPP III is much shorter (558 amino acids) than the already reported human (737 amino acids) [54], yeast (711 amino acids) [55] and *B*. *thetaiotaomicron* DPP III (675 amino acids) [19]. Similarly to the other available crystal structures of M49 family enzymes, the structure of *Ca*DPP III consists of two structural domains, called upper (containing zinc ion) and lower domain, which are separated by the inter-domain cleft. The zinc ion, essential for the enzyme activity, is positioned in the lower part of the upper domain where it is pentacoordinated by two histidines from the first conserved, zinc binding, motif H^379^EISH, one glutamic acid (E442—bidentately) from the second motif E^441^ECKAD and a water molecule. Although *Ca*DPP III has shorter first conserved motif, pentapeptide *vs* hexapeptide, there is no significant difference in the zinc binding site in these two orthologs (Fig 2). Although smaller than the other DPPs III, the *Ca*DPP III lower structural domain core is also comprised of five-stranded β-barrel surrounded by α-helixes.

**Fig 3:**
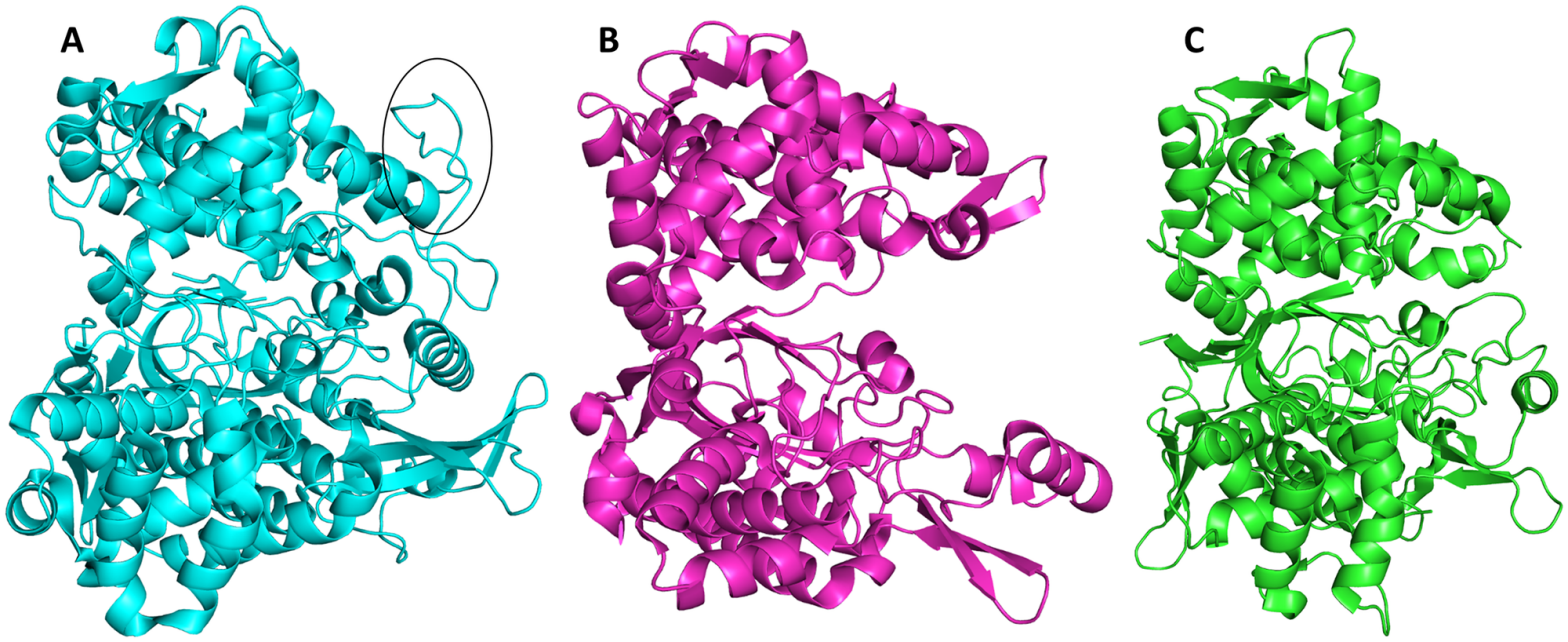
Cartoon representation of several DPP III structures determined by X-ray diffraction (A) h.DPP III in open conformation (PDB ID: 5E33), (B) *Ca*DPP III (PDB ID: 6EOM) and (C) *Bt*DPP III in closed conformation (PDB ID: 5NA8). The long loop between two conserved, zinc binding, motifs present in h.DPP III is encircled. The figure was prepared using the PyMol program [51].

**Fig 4:**
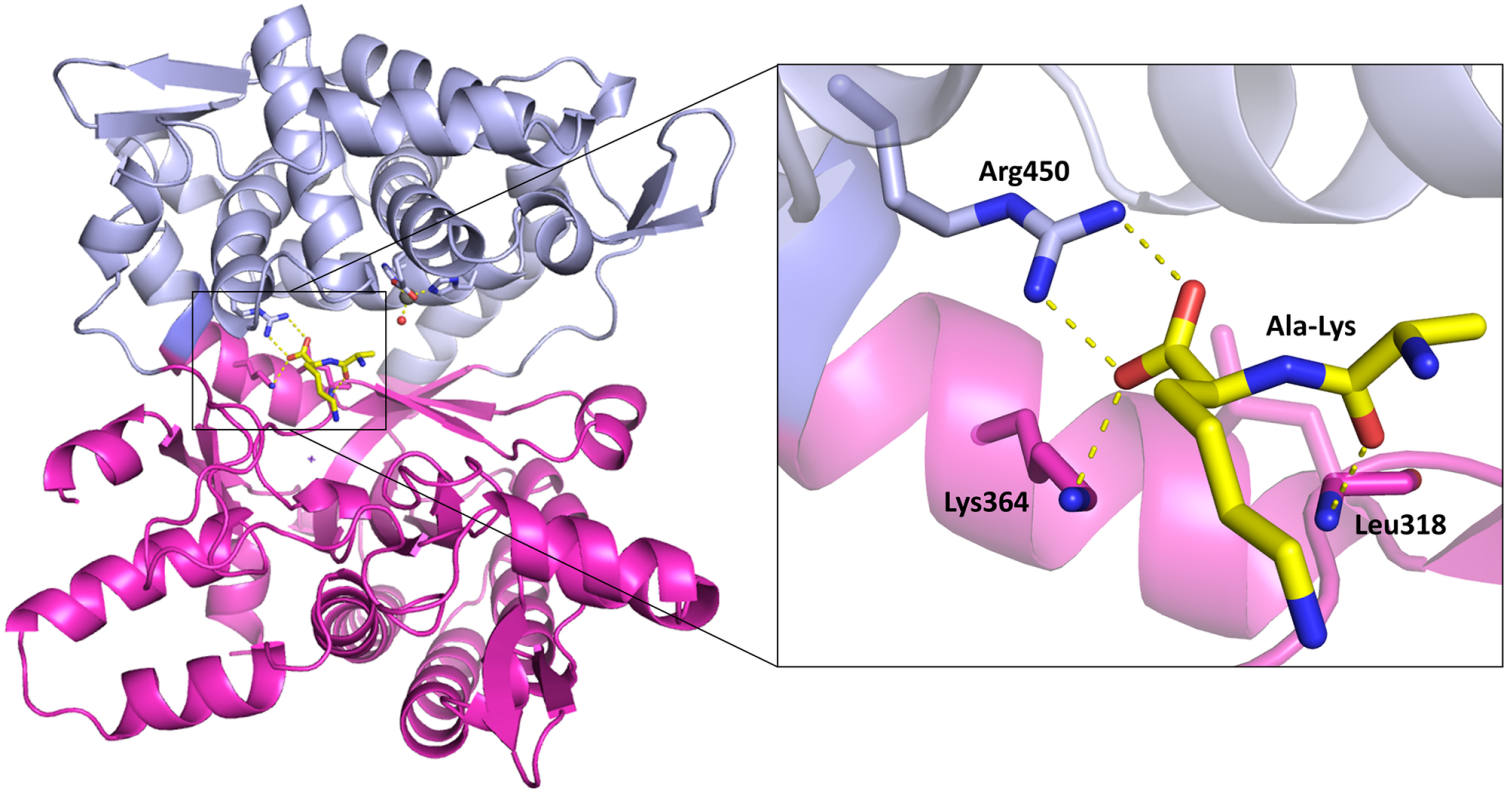
Dipeptide Ala-Lys bound to the *Ca*DPP III protein. Upper structural domain is shown in blue and lower in magenta. Dipeptide Ala-Lys is shown as yellow sticks. The figure was prepared using the PyMol program [51].

The long range conformational changes from open to closed form were determined for all up to date characterized DPPs III [19, 54, 56], wherein the ligand binding boosts the protein closure. The crystal structure of *Ca*DPP III is very compact and more similar to the closed *B*. *thetaiotaomicron* and human DPP III forms than to the open ones (Figs 2 and 3). The conformation of *Ca*DPP III is partially closed, probably due to the Ala-Lys dipeptide bound into the protein active site. Since Ala-Lys was never added to the crystallization solution, we assume that it was bound into the inter-domain cleft during protein expression. The peptide is located deeply inside the cleft and occupies S1’ and S2’ subsites (see its alignment with the structure of the human DPP III—tynorphin complex, PDB_code 3T6B, S3 Fig). In this position it is stabilized by four hydrogen bonds: two with Arg450 (2.8 and 3.1 Å) from the upper domain, and one with each Lys346 (2.7 Å) and Leu318 (2.8 Å), both from the lower domain (Fig 4).

Due to the significant difference in *Bt*DPP III and *Ca*DPP III conformations, we divided *Ca*DPP III into an upper and lower domain, which were subsequently treated as separate objects. Superposition of the upper domain of *Ca*DPP III (264–298 and 350–541) with the corresponding upper domain of *Bt*DPP III gave rise to an RMSD value of 1.6 Å. An analogous alignment of the respective lower domains of *Ca*DPP III (32–263, 299–349 and 542–555) and *Bt*DPP III yielded an RMSD value of 1.5 Å. All secondary structure elements present in the *Bt*DPP III upper domain were also found in the upper domain of *Ca*DPP III. However, the lower *Ca*DPP III domain is 85 amino acid residues shorter and lacks the α-helix-loop-α-helix motif, two β-strands, and α-helix at C-terminus (S4 Fig). Unlike yeast and human, bacterial DPPs III do not have the long loop between two conserved, zinc binding, motifs (Fig 3).

In order to examine the structural basis for the increased thermal stability of *Ca*DPP III, we compared the secondary structures and interactions within the DPP III enzyme from a mesophile *B*. *thetaoitaomicron* and thermophile *C*. *abyssi* (Table 4). The potential stabilizing interactions were determined using the Arpeggio server [57], considering only the residues in the crystal structures (647 and 524 residues for *Bt*DPP III and *Ca*DPP III, respectively). The higher ratios of α –helices and β –sheets seen in *Ca*DPP has previously been connected with increased thermal stability [58, 59]. The increased abundance of proline, known to reduce the flexibility of the main chain, has also been noticed [60]. Previous studies have shown higher residue hydrophobicity, fewer polar residues and increased amount of charged nonpolar residues in thermophiles [61], all of which have been observed in *Ca*DPP III when compared with its mesophile counterparts. Apparently, the higher relative number of non-covalent interactions within the protein the larger is stability of the structure [61–63]. In the case of *Ca*DPP III it is manifested by enhanced thermal stability.

**Table 4:** Structural factors comparison between the termophile *C*. *abyssi* and mesophile *B*. *thetaiotaomicron*.

	*Bt*DPP III		*Ca*DPP III		Relative occurence**	
	Abs.	Rel. (%)	Abs.	Rel. (%)	*Bt* (%)	*Ca* (%)
**Residues in α—helix**	374	57.80	332	63.36	91.2	100
**Residues in β—sheet**	86	13.29	82	15.65	84.9	100
**Proline residues**	23	3.4	26	4.7	72.3	100
**Polar residues**	122	18.0	87	15.6	100	86.7
Charged residues*	130	19.3	130	23.3	82.8	100
**Hydrophobic residues**	288	42.7	247	43.7	97.7	100
**Hydrogen bonds**^a^	741	1.145	606	1.156	99.1	100
**Weak hydrogen bonds**^b^	502	0.776	431	0.823	94.3	100
**Ionic interactions**^c^	94	0.145	92	0.176	82.4	100
**Hydrophobic contacts**^c^	1814	2.804	1614	3.080	91.0	100
**Aromatic contacts**	127	0.196	113	0.216	90.7	100

^a^Cut-off value: 3.9 Å, *ϴ* > 90°;

^b^Value: 3.6–3.9 Å, 90° > *ϴ* > 130°;

^c^Cut-off value: 4 Å

*Glu, Arg, Lys

**Scaled to the highest value

### Computational study

According to our previous study on human DPP III we consider the *d*_1_ and *d*_2_ (Cα distances between E142-K404 and E330-K404, respectively) distances as a measure of the protein compactness and mutual orientation of the two domains [47]. The conformers with similar *d*_1_ and *d*_2_ values are considered to belong into the same class. The existence of the inter-domain cleft in the experimentally determined structure, WT_E_ (with the *d*_*1*_ distance of 22.6 Å), large enzyme promiscuity, and the presence of two of the five highly conserved regions in the lower protein domain, suggest that *Ca*DPP III could experience long-ranged inter-domain motion. Such motion has been previously observed in human, *B*. *thetaiotaomicron* and yeast orthologs as well.

In order to thoroughly investigate the possible long range conformational changes of *Ca*DPP III, and to find out how dipeptide-2-naphthylamides influence these changes, we performed a series of MD simulations of both ligand free *Ca*DPP III and its complexes with Arg_2_-2NA, Gly-Arg-2NA, Gly-Phe-2NA and Gly-Pro-2NA. Further on, we simulated the HEISH → HEISGH mutant and its complex with Arg_2_-2NA to study influence of this conserved motif on the enzyme dynamics and the active site structure as well as on ligand binding.

#### MD simulations of the ligand free *Ca*DPP III, WT and the HEISGH mutant

**Fig 5:**
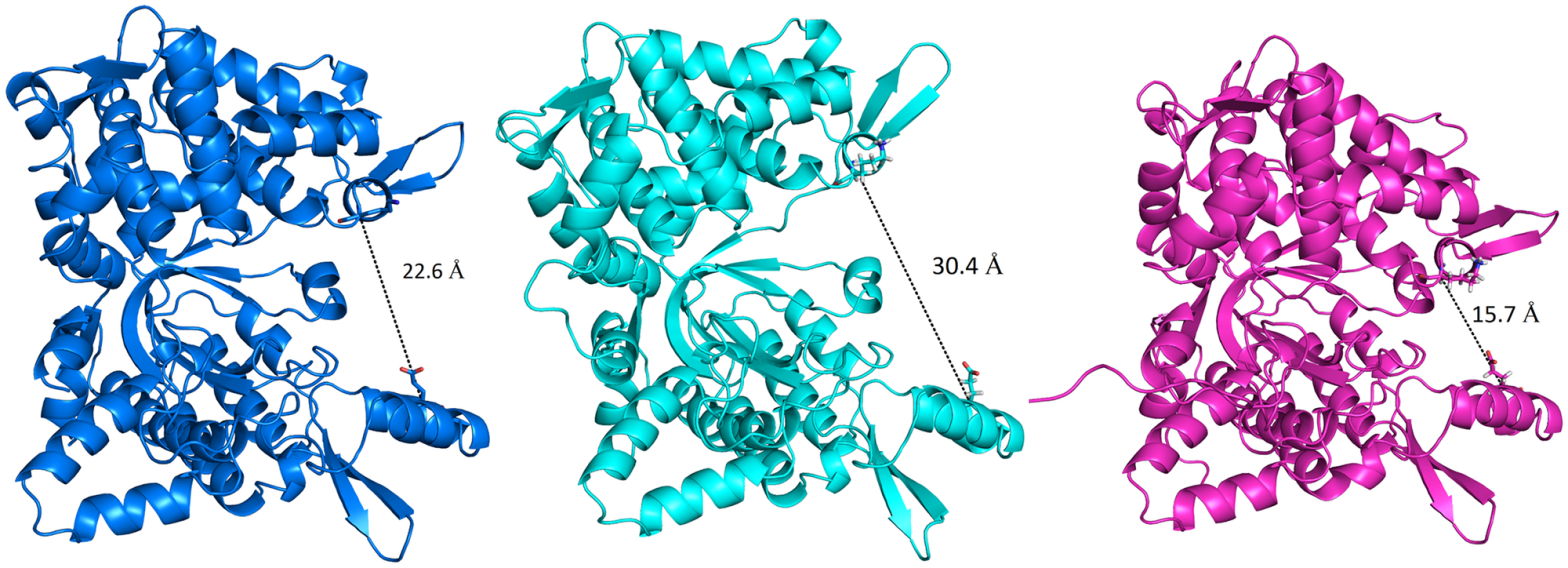
Three representative, the most distinct, forms (conformers) of *Ca*DPP III: crystal, WT_E_ (left), open, WT°AMD, (middle) and closed, WTcMD (right) obtained from accelerated and conventional MD simulations. Distance d_1_ (E142 –K404) is shown as the black dashed line. The figure was prepared using the PyMol program [51].

The MD simulations starting from the crystallographically determined structure of *Ca*DPP III revealed long-range conformational changes corresponding to the inter-domain motion (Fig 5), described as protein opening and closing. Interestingly, the significant inter-domain motion was observed already during the equilibration. The *d*_*1*_ distance decreased from 22.6 to 15.4 Å, and the protein transformed from WT_E_ to a more compact, so called WT^c^_EQ_, form. During the productive MD simulations, the *Ca*DPP III structure reopened and transformed to an extended form first, and then again to the compact, WT^C^_MD_ one (Fig 5 and S5 Fig).

Geometrical values describing the most representative structures obtained during MD simulations are listed in S6 and S7 Tables. Apparently, both compactness and mutual orientation of the experimental and the simulated structures differ (see S6 and S7 Tables), so we considered them as distinct forms of *Ca*DPP III. The most compact structure of the ligand free protein, WT^c^_EQ_, was obtained immediately after the equilibration phase, while the most extended structure, WT^**°**^_AMD_, was obtained after 40 ns of aMD simulations (Fig 5). Both types of simulations started from the crystallographically determined structure. It should be noted that variation of *d*_*1*_ (a measure of the protein compactness) during MD simulations of *Ca*DPP III is smaller than variations determined for human [64] and *B*. *thetaiotaomicron* orthologs [50] (Δd ≈ 8 Å in *Ca*DPP III, 17 Å in h.DPP III and 16 Å in *Bt*DPP III).

The MM-PBSA energies calculated for the 5 ns intervals along the trajectories are shown in S6 Fig. Since MM-PBSA energy mostly represents the enthalpy of the system it could be stated that the compact *Ca*DPP III conformers have larger enthalpies than the extended one (change of the structure compactness during MD simulations is shown in S5 Fig). Similar changes of the system enthalpy were determined for the human DPP III and *Bt*DPP III as well [50, 64]. However, the enthalpy difference between two forms is compensated by the solvent entropy increase due to expulsion of water molecules from the confined, inter-domain cleft region upon protein closure. Namely, the number of waters trapped in the inter-domain cleft is about 90 in the open and about 40 in the closed enzyme form. According to the energy values for a water molecule release quoted in literature (from 1.2 to 2.3 kcal mol^-1^ [65, 66]) the transformation from the WT^**°**^_MD_ form to WT^c^_MD_ results in a 60 to 115 kcal mol^-1^ change in energy due to water molecule expulsion and approximately compensates the enthalpy increase.

Although the protein compactness and mutual orientation of two domains changed during the simulations, conformation of each domain itself remained unchanged, for example *RMSD* between domains in WT_E_ and WT^c^_MD_ is 0.77 Å and 0.94 Å for the upper and lower domain, respectively.

During the simulations Zn^2+^ was coordinated by two histidines (His379 and His383) and Glu412, either monodentately or bidentately (S7 and S8 Figs). These residues belong to the conserved regions of the DPP III family, H^379^EXGH^383^ and E^411^ECR(K)A^415^ [10]. Differently from the crystal structure, which contains one water molecule in the zinc ion coordination sphere, in the structures obtained by MD simulations the zinc ion is coordinated with up to three water molecules (S9 Fig). These water molecules frequently exchange with ‘bulk’ water indicating relatively fast inter-domain motions. Such rapid long range domain motions have not been traced neither during the simulations of human nor *Bt*DPP III [50, 64].

The Zn^2+^ coordination, with two histidines and the second Glu from the E^411^ECR(K)A^415^ region is, according to our previous quantum mechanical studies on human DPP III, required for the enzymatic reaction [67].

MD simulations of the ligand-free mutant with the HEISGH hexapeptide (present in human and other characterized bacterial DPPs III) instead of the pentapeptide HEISH motif have been performed in order to elucidate possible influence of the pentapeptide with hexapeptide motif replacement on the protein structure and dynamics. The structure obtained after the energy minimization and equilibration was even more compact than in the case of the wild-type enzyme (*d*_*1*_ 11.1 Å and 15.4 Å, respectively). This mutation also affected the zinc ion coordination. During the equilibration of the HEISGH mutant Glu380 entered the coordination sphere acting as a bidentate ligand for the first ~35 ns, and as a monodentate ligand for the rest of the simulation time (S10 and S11 Figs). Such zinc ion coordination was also noticed during the MD simulations of human [68] and *B*. *thetaiotaomicron* [50] DPP III orthologs, both containing the HEXXGH signature motif.

Further on, during MD simulations of the wild-type enzyme, conformational changes corresponding to the domain closing, opening and again closing were observed, while the HEISGH mutant after reopening did not close again (S6 Table) within the simulated timeframe (S12 Fig).

#### MD simulations of the *Ca*DPP III complexes with substrates

In order to understand the effect of ligand binding on the degree and rate of protein closure, as well as to try to explain the measured difference in substrate specificity, *Ca*DPP III complexes with Arg_2_-2NA, Gly-Arg-2NA, Gly-Phe-2NA and Gly-Pro-2NA were simulated for 150 ns each. Since recent simulations of the human and bacterial DPP III—Arg_2_-2NA complexes had shown that Arg_2_-2NA forms strong and persistent interactions with the binding site when the enzyme is in the more compact form [50, 64], the compact enzyme structure, WT^C^_EQ_ (S7 Table), obtained after the equilibration was used for docking. The MD simulations showed that the conformational change corresponding to the protein closure is even more pronounced in the complexes than in the ligand-free enzyme. Namely, *d*_*1*_ (E142 –K404 distance) is about 15 Å in the most compact ligand-free enzyme, while it is about 11 Å in complexes with Arg_2_-2NA and Gly-Arg-2NA (S8 Table) and about 12 Å in complex with Gly-Phe-2NA. On the other hand no significant change in the protein tertiary structure was observed during the simulations of the *Ca*DPP III—Gly-Pro-2NA complex, the weakest substrate of all dipeptidyl-2-naphthylamides tested in this study (Table 2). It must be noticed that during MD simulation substrates remained close to their initial binding positions, i.e. they remained bound in the form of a β-strand antiparallel to the five-stranded β-core from the lower protein domain.

The MM-GB and MM-PB energies are only a crude approximation of the enthalpic component of binding free energies (Table 5). But, since the ligands considered in this study are closely related in size, the changes in entropy upon complexation should not significantly influence the relative binding affinities. So, from the relative MM-GB and MM-PB energies we can say that Arg_2_-2NA is better substrate of *Ca*DPP III than Gly-Arg-2NA, as well as that Gly-Phe-2NA and Gly-Pro-2NA are poor *Ca*DPP III substrates, in accord with the experimentally determined kinetic data.

**Table 5:** The MM-GB and MM-PB binding energies calculated for the *Ca*DPP III complexes with Arg_2_-2NA, Gly-Arg-2NA, Gly-Phe-2NA and Gly-Pro-2NA. Energies are given in kcal mol^-1^.

Ligand	cMD		aMD*	
	ΔE_MM-PB_	ΔE_MM-GB_	ΔE_MM-PB_	ΔE_MM-GB_
**Arg**_**2**_**-2NA**	-86.0 (±9.8)	-103.4 (±7.1)	-89.0 (±10.6)	-106.6 (±7.6)
**Gly-Arg-2NA**	-70.6 (±11.5)	-89.5 (±6.9)	-60.5 (±5.2)	-79.2 (±6.2)
**Gly-Phe-2NA**	-19.3 (±6.0)	-72.8 (±6.5)	-22.6 (±7.7)	-75.1 (±6.4)
**Gly- Pro-2NA**	-21.2 (±6.4)	-72.7 (±6.4)	-18.6 (±7.0)	-67.5 (±6.3)

*aMD simulations were performed for 50 ns starting from the final structure of cMD simulations

**Fig 6:**
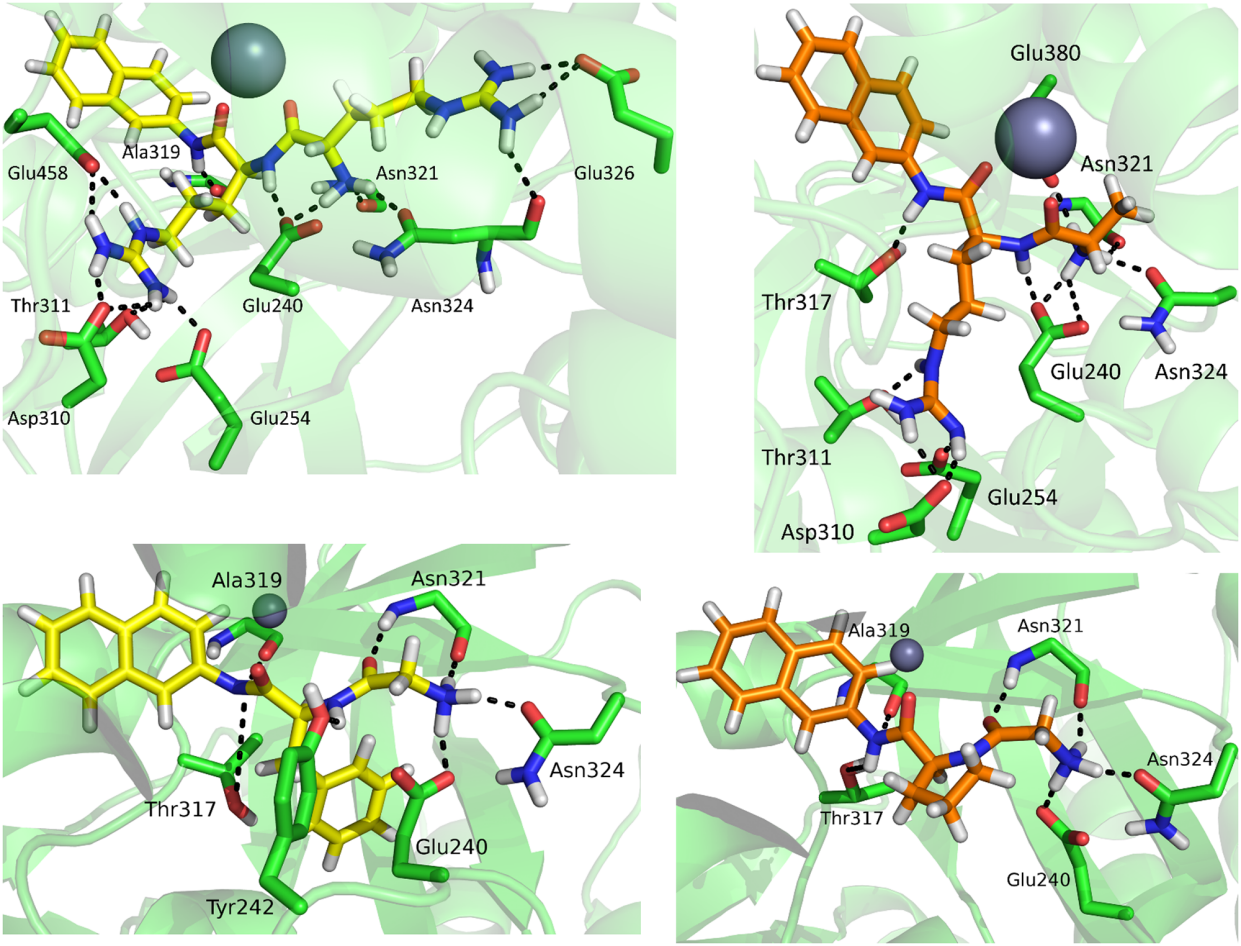
Interactions between substrates, Arg_2_-2NA (up, left), Gly-Arg-2NA (up, right), Gly-Phe-2NA (down, left) and Gly-Pro-2NA (down, right) and the amino acid residues from the *Ca*DPP III active site. The figure was prepared using the PyMol program [51].

The relative enzyme activity towards different dipeptidyl-2-naphthylamides (Table 2) can also be rationalized by the substrate orientation in the enzyme active site, and by the hydrogen bond analysis (Table 6). Apparently, Arg_2_-2NA has formed more hydrogen bonds during MD simulations than Gly-Arg-2NA (Fig 6). Further on, in the *Ca*DPP III—Arg_2_-2NA complex the naphthalene is slightly more buried in the hydrophobic pocket than in the *Ca*DPP III—Gly-Arg-2NA complex. The protein—ligand interactions determined from the computational study are in agreement with the location of S1 and S2 subsites proposed on the basis of the *Ca*DPP III alignment with h.DPP III and *Bt*DPP III (S9 Table) [69].

**Table 6:** Hydrogen bonds population (%). The analysis was performed for the lowest-energy 5 ns long fragments of the 150 ns long (100 ns cMD + 50 ns aMD) trajectories used to calculate the MM-PBSA energies. The hydrogen bonds occurring <5% in all of the sampled structures are omitted.

Acceptor	Arg_2_-2NA	Gly-Arg-2NA	Gly-Phe-2NA	Gly-Pro-2NA
Glu240	194.8	208.4	123.9	104.0
Tyr242	-	-	39.8	-
Glu254	90.8	87.6	-	-
Asp310	130.0	136.4	-	-
Thr311	17.6	39.2	-	-
Val315	-	13.6	-	-
Thr317	-	98.0	-	14.3
Ala319	90.0	-	12.0	-
Asn321	112.0	48.4	97.2	95.2
Asn324	104.4	65.6	86.9	83.3
Glu326	147.2	-	-	-
Glu380	-	64.4	-	-
Glu411	6.0	-	-	-
Glu458	163.2	-	-	-
Donor	Arg_2_-2NA	Gly-Arg-2NA	Gly-Phe-2NA	Gly-Pro-2NA
Glu240	-	14.0	-	-
Val315	-	5.2	-	-
Thr317	8.8	-	-	-
Asn321	23.2	31.2	84.1	59.0

In both orthologs Arg_2_-2NA is stabilized with the strong electrostatic interactions between the Arg backbone carbonyl and the zinc ion, as well as through the number of hydrogen bonds and electrostatic interactions with the amino acid residues from the enzyme subsites S1 (Glu254, Asp310 and Glu458) and S2 (Glu240, Asn321, Asn324 and Glu326) during the MD simulations. Further on, its N-terminus and the first carbonyl group are stabilized by Glu240 (Glu316 in h.DPP III; Glu307 in *Bt*DPP III), Asn321 (Asn391 in h.DPP III; Asn385 in *Bt*DPP III) and Asn324 (Asn394 in h.DPP III; Asn388 in *Bt*DPP III) while the side chains of substrate arginines interact with Glu240 (Glu316 in h.DPP III; Glu307 in *Bt*DPP III), Glu254 (Glu329 in h.DPP III; Glu320 in *Bt*DPP III) and Glu326 (Asp396 in h.DPP III; Asn390 in *Bt*DPP III) (S13 Fig). It should be noted that, although the number of the negatively charged amino acid residues is higher in the substrate binding site of *Ca*DPP III than of h.DPP III and *Bt*DPP III (S11 Table), the *K*_*M*_ value (which we could consider as a crude approximation of binding affinity) is about one order of magnitude higher for binding of Arg_2_-2NA to *Ca*DPP III than to *Bt*DPP III (and h.DPP III). We assume that the main reason for this is the smaller width of the inter-domain cleft of the previous, which results with higher rigidness of the substrate binding site in *Ca*DPP III. So, positioning of the hydrophobic naphthylamide core within such a limited, mostly negatively charged region (except the so called hydrophobic pocked which is deeply buried) is energetically unfavorable.

Gly-Phe-2NA and Gly-Pro-2NA form significantly less hydrogen bonds with the enzyme during MD simulations than Arg_2_-2NA and Gly-Arg-2NA in accord with the measured enzyme activity towards these substrates. While Arg_2_-2NA and Gly-Arg-2NA coordinate the Zn^2+^ ion with both carbonyl oxygens, Gly-Phe-2NA and Gly-Pro-2NA coordinate it only with the second carbonyl group. Their first carbonyl group from the N-terminus makes a hydrogen bond with Asn321. N-termini itself makes hydrogen bonds with Glu240, Asn321 and Asn324. The substrates’ amide groups occasionally make electrostatic interactions or hydrogen bonds with Ala319. Side chains of the phenylalanine and proline residues are stabilized by Van der Waals interactions with amino acids from the lower protein domain: Tyr242, Phe256, Thr311, Thr317, Phe320 and Leu322.

#### MD simulations of the HEISGH *Ca*DPP III mutant complex with Arg_2_-2NA

**Fig 7:**
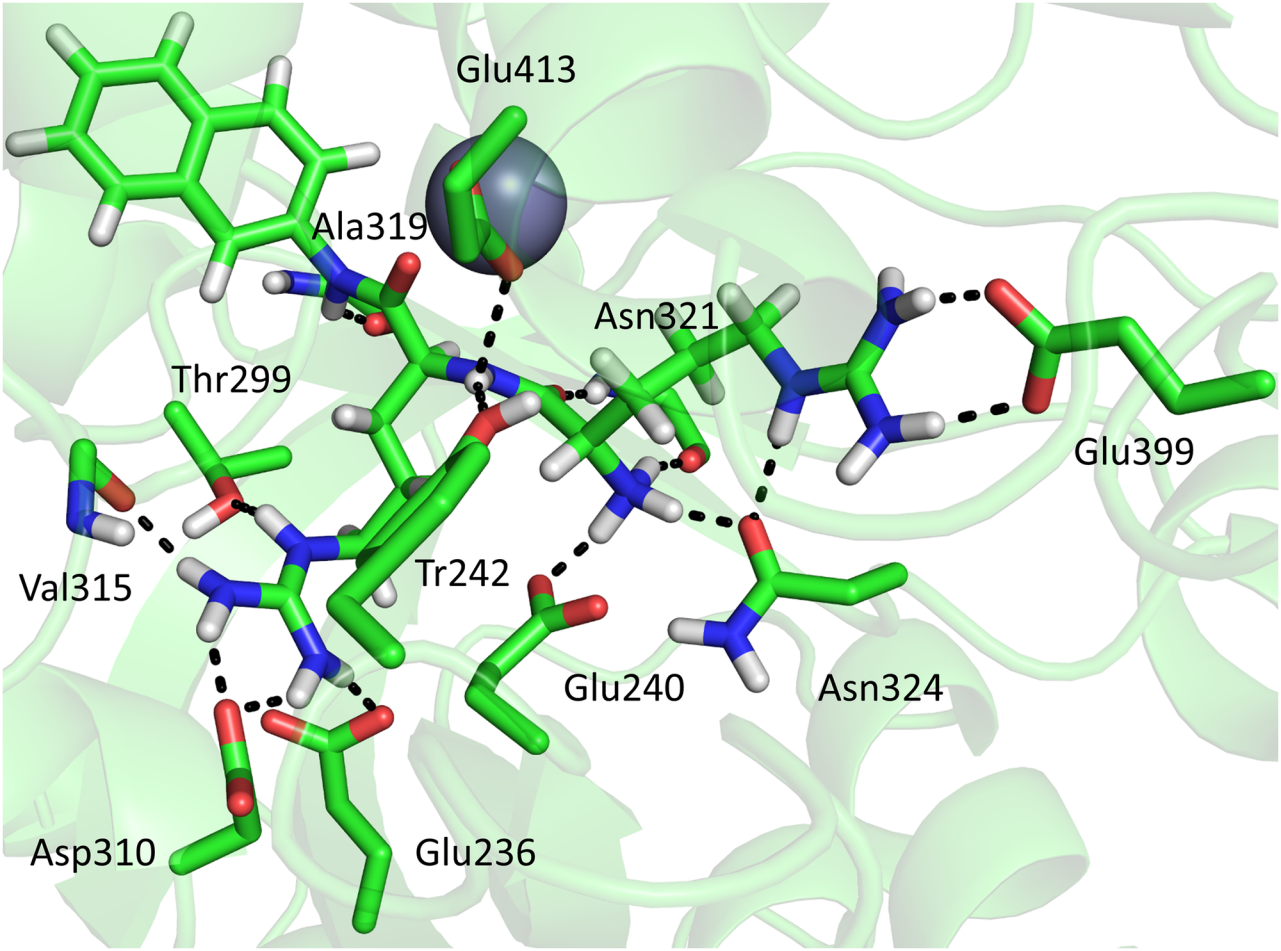
Interactions between Arg_2_-2NA and the amino acid residues in the binding site of the HEISGH *Ca*DPP III mutant. The figure was prepared using the PyMol program [51].

The Arg_2_-2NA positioning in the HEISGH mutant differs slightly from that in the wild type enzyme (Figs 6 and 7). In wt-*Ca*DPP III both carbonyl atoms of the Arg_2_-2NA backbone enter the Zn^2+^ coordination sphere. In the complex with HEISGH mutant only the first carbonyl atom coordinates Zn^2+^. This could be explained by different positioning of the second Arg residue caused by its interactions with Glu413, whose position was shifted due to the pentapeptide to hexapeptide mutation. The hydrogen bonds with Glu240, Glu254, Asp310, Ala319, Asn321 and Asn324 found in the complex with the wild-type enzyme are preserved in the complex with mutant, as well. Additionally, in the complex with the wild-type enzyme, three hydrogen bonds are formed with Thr311, Glu326 and Glu458, while in the complex with the mutant the substrate is hydrogen bonded with Tyr242, Val315, Thr317 and Glu399 (S10 Table). The MM-PBSA calculations suggest that Arg_2_-2NA binds weaker to the HEISGH mutant than to the wild type enzyme (-89.0±10.6 *vs* 81.9±11 kcal mol^-1^).

#### Investigations of the *Ca*DPP III ligand site polarity

In order to investigate polarity of the ligand-binding site in *Ca*DPP III, we performed short MD simulations with tynorphin bound into its active site in the same orientation as it is bound in the crystal structure of human DPP III—tynorphin complex (PDB code: 3T6B). The population of charged amino acid residues in the protein region around 6 Å of tynorphin determined in human DPP III, *Bt*DPP III and *Ca*DPP III revealed that the number of negatively charged amino acids is higher in *Ca*DPP III than in the other two, while the number of positively charged amino acid residues is smaller than in human, but larger than in *Bt*DPP III (S11 Table). In summary, the ligand binding site in *Ca*DPP III is more negative than the binding sites in the other two orthologs.

## Conclusions

This work presents results of biochemical and structural characterization of *Ca*DPP III, the first enzyme from the M49 family isolated from a thermophilic organism. Furthermore, this is the first functionally and structurally characterized member of this family with hexapeptide M49 signature motif reduced to the pentapeptide HEISH.

With the sequence length of about 550 amino acid residues *Ca*DPP III is much smaller than the other M49 peptidases characterized up to date (675 to 738 amino acids). Its structure, stability and flexibility were determined by X-ray diffraction and molecular dynamics simulations. Its overall structure and the zinc coordination are similar to that of its mesophilic counterparts despite difference in the overall size and the active site motif. Interestingly, the fluctuations of *Ca*DPP III domains are faster than those determined for the human and *B*. *thetaiotaomicron* orthologs. However, the range of inter-domain cleft opening is smaller pointing to the decreased plasticity of its inter-domain active site in comparison to the other up to date characterized DPPs III. The finding that the relative number of non-covalent interactions within *Ca*DPP III is larger, while the share of loops (unstructured regions) is lower than in its mesophilic counterparts suggests higher rigidity and compactness of this enzyme, and gives possible explanation for its thermal stability.

The study of *Ca*DPP III catalytic performances was performed on a set of dipeptide-2-naphthylamides. Differently from the other characterized members of the DPP III family which show high substrate specificity towards Arg_2_-2NA, *Ca*DPP III has similar substrate specificity towards several dipeptide-2-naphthylamides. According to our previous findings regarding the zinc ion coordination and the mechanism of DPP III catalyzed peptide bond hydrolysis, it seems that the difference in size and polarity of the active site as well as of the conserved, zinc binding motif, does not affect catalytic role of the metal ion in *Ca*DPP III, *i*.*e*. it is appropriate for the enzymatic reaction. The possible explanation for the decrease in *Ca*DPP III activity and specificity towards dipeptide-2-naphthylamide substrates could be found in the binding site potency to accommodate these substrates. In line with this are the measured kinetic parameters, i. e. *Ca*DPP III has an order of magnitude higher *K*_*M*_ value for the preferred substrate Arg_2_-2NA than *Bt*DPP III. There are several structural characteristics of *Ca*DPP III that could be reason for this, like the more compact enzyme structure and less adaptable and more negative active site, in comparison with its mesophilic counterparts, which hinder binding of the bulky, hydrophobic naphthalene ring. Further on, the fast, low amplitude alternation between the open and closed form of *Ca*DPP III might also be limiting factor for binding of the substrate in catalytically active orientation. In summary, we could conclude that the measured decrease in activity and substrate specificity is correlated with higher polarity and lower plasticity of the active site in *Ca*DPP III with respect to the other DPP III orthologs.

## Supporting information

S1 Fig SignalP scores for the first 70 residues- C-score is a raw cleavage site score, S-score is a signal peptide score, Y-score is a combined cleavage site score (geometric average of the C-score and slope of the S-score). (TIF)

S2 Fig Thermal inactivation of *Ca*DPP III. Relative activity compared to the highest residual activity measured after the incubation of the enzyme at 50 °C.
(TIF)

S3 Fig Structure of the *Ca*DPP III active site superpositioned on the human DPP III—Tynorphin complex (PDB_code 3T6B). Lys-Ala dipeptide is shown with green sticks; tynorphin is shown with cyan sticks. Zinc ion is represented by a grey sphere.
(TIF)

S4 Fig Separately superimposed upper and lower domains of *Ca*DPP III (magenta) on *Bt*DPP III (green). *Ca*DPP III lacks α-helix-loop-α-helix motif (left) and two β-strand and α-helix (right). Missing motifs are marked with black ellipses.
(TIF)

S5 Fig The selected Cα atom distance profiles obtained from cMD and aMD simulations of the wild type enzyme. (TIF)

S6 Fig Relative MM-PBSA energies calculated for the wild-type enzyme during 200 ns of MD simulations of *Ca*DPP III. The values determined for the sets of structures sampled during 5 ns intervals in raw are shown.
(TIF)

S7 Fig The representative Zn^2+^ coordination in the wild-type enzyme: (left) with Glu380 monodentate and (right) bidentate ligand of Zn^2+^ during the MD simulations. Zinc ion is shown as a grey sphere.
(TIF)

S8 Fig Distance between Zn^2+^ and the carboxyl oxygens of the coordinating glutamates, Glu380 (left) and Glu412 (right) residues during cMD-ff14SB simulation of the wild-type *Ca*DPP III. (TIF)

S9 Fig “Resistance time” for the water molecules coordinating Zn^2+^ in *Ca*DPP III during 200 ns of MD simulations of the ligand free enzyme. Water molecules appearing in less than 200 sampled frames are omitted.
(TIF)

S10 Fig The representative Zn^2+^ coordination in the HEISGH mutant: (left) with Glu412 monodentate and (right) bidentate ligand of Zn^2+^ during the MD simulations. Zinc ion is shown as a grey sphere.
(TIF)

S11 Fig Distance between Zn^2+^ and the carboxyl oxygens of coordinating Glu380 (left) and Glu412 (right) residues during cMD-ff14SB simulation of the *Ca*DPP III HEIGSH mutant. (TIF)

S12 Fig Radius of gyration (left) and *d*_1_ distance (right) for the wild-type enzyme and the HEISGH mutant during 200 ns of MD simulations. (TIF)

S13 Fig Active sites of DPP III in the complex with Arg_2_-2NA: *Ca*DPP III (protein shown in green and substrate in magenta) and h.DPP III (protein shown in cyan and substrate in yellow). Zn^2+^ is shown as a grey sphere.
(TIF)

S1 Table The average energy values determined during the first 50 ns of MD simulation of the ligand-free enzyme using ff14SB and the parameters required for aMD simulations. All values are given in kcal mol^-1^.
(DOCX)

S2 Table The average energy values determined during the 50 ns of MD simulations of *Ca*DPP III—RRNA complex using ff14SB and the parameters required for aMD simulations. All values are given in kcal mol^-1^. *E*_r_ and *E*_a_ values are 1.0 and 0.1 kcal mol^-1^, respectively.
(DOCX)

S3 Table Relative activity of *Ca*DPP III at pH 6–7 in 50 mM phosphate buffer, and pH 7–8.6 in 50 mM Tris HCl buffer at 37 and 50 °C. (DOCX)

S4 Table The influence of effectors on *Ca*DPP III activity towards Arg_2_-2NA and Gly-Arg-2NA substrates. (DOCX)

S5 Table Influence of metal ions on *Ca*DPP III peptidase activity towards Arg_2_-2-NA substrate measured at 50°C and pH 7.0. (DOCX)

S6 Table Values of the geometric parameters used to describe degree and type of *Ca*DPP III closure determined in the most distinct enzyme structures, experimental and those obtained using conventional MD simulations. The radius of gyration (*R*_g_) was calculated for the protein backbone atoms. The distances *d*_1_ and *d*_2_ were calculated for Cα atoms. *RMSD* calculated for lower (*RMSD*_LD_) and upper domain (*RMSD*_UD_) with respect to the experimentally determined structure is given in the last two rows.
(DOCX)

S7 Table Values of the geometric parameters used to describe degree and type of *Ca*DPP III closure determined in the most distinct enzyme structures, experimental and those obtained using accelerated MD simulations. The radius of gyration (R_g_) was calculated for the protein backbone atoms. The distances *d*_1_ and *d*_2_ were calculated for Cα atoms. RMSD calculated for lower (*RMSD*_LD_) and upper domain (*RMSD*_UD_) with respect to the experimentally determined structure is given in the last two rows.
(DOCX)

S8 Table Geometric parameters determined for the *Ca*DPP III complexes with Arg_2_-2-NA, Gly-Ala-2-NA, Gly-Phe-2-NA and Gly-Pro-2-NA during 150 ns of MD simulations. Values of representative, the lowest energy structures* are given.
(DOCX)

S9 Table Amino acid residues composition of the S1 and S2 subsites in the *Ca*, *Bt*, yeast and human DPPIII. The non-conserved amino acid residues are given in bold.
(DOCX)

S10 Table Hydrogen bonds population (%) for the HEISGH mutant complex with Arg_2_-2NA. The analysis was performed for the lowest-energy 5 ns long fragments of the 150 ns long (100 ns cMD + 50 ns aMD) trajectories used to calculate the MM-PBSA energies. The hydrogen bonds occurring <5% in all of the sampled structures are omitted.
(DOCX)

S11 Table Number of charged amino acid residues within 6 Å of tynorphin bound into the enzyme active site. (DOCX)
