# Supplementary figures and images for: The first dipeptidyl peptidase III from a thermophile: Structural basis for thermal stability and reduced activity
Source: PLoS One. 2018 Feb 8;13(2):e0192488. doi: 10.1371/journal.pone.0192488 (PMC5805324; doi:10.1371/journal.pone.0192488)

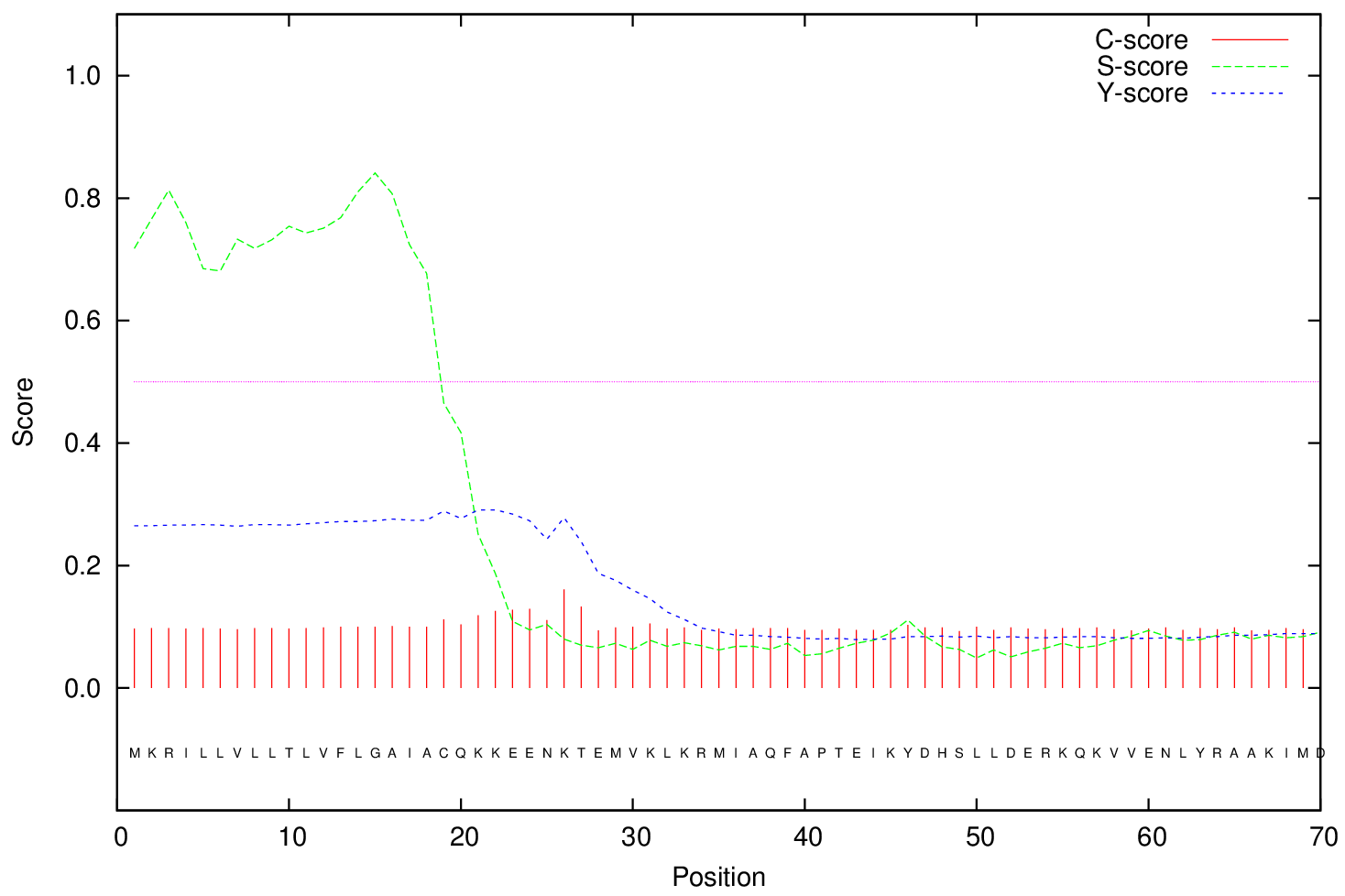

Supplement: S1 Fig — (TIF) [file pone.0192488.s001.tif]

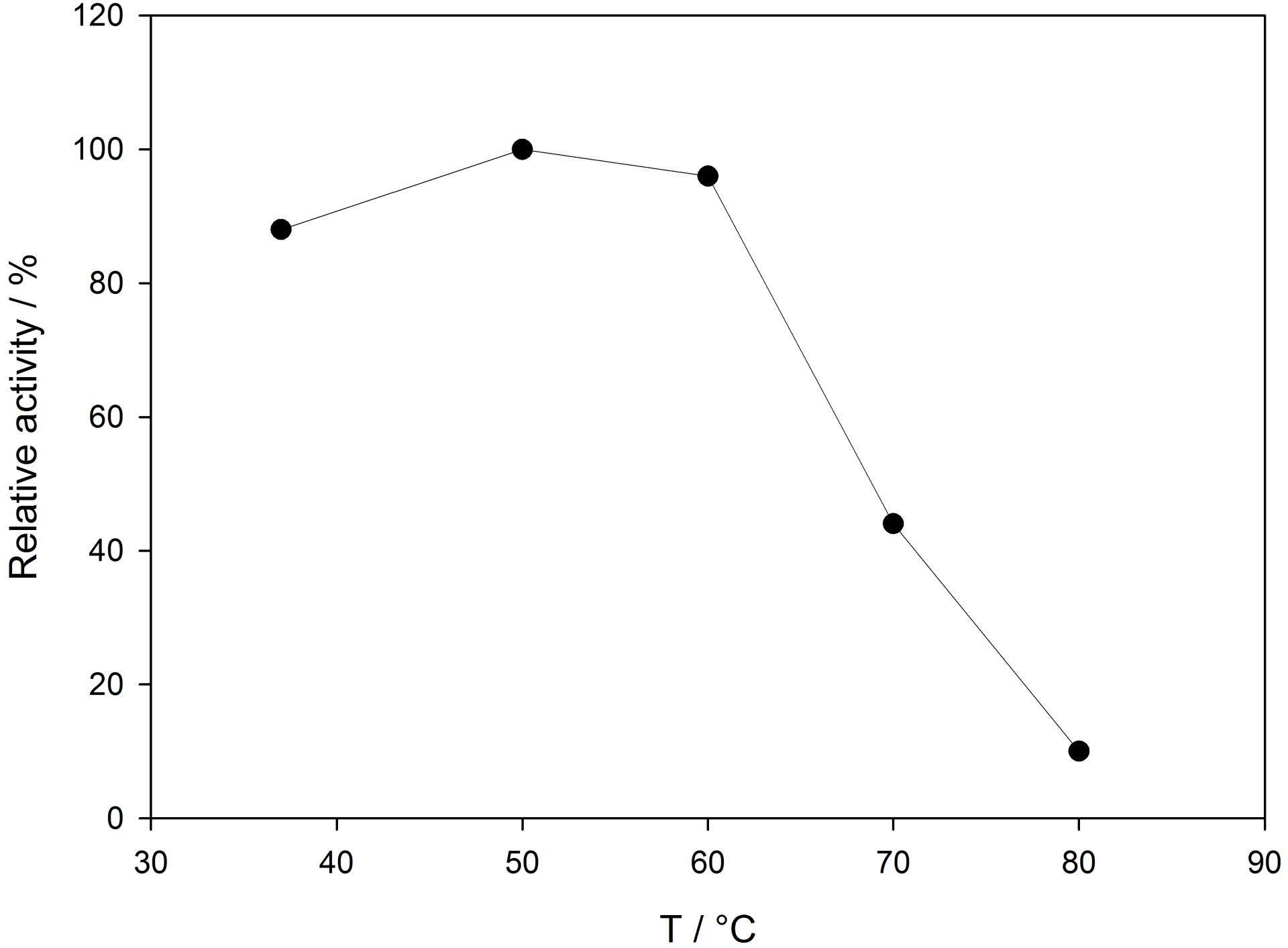

Supplement: S2 Fig — Relative activity compared to the highest residual activity measured after the incubation of the enzyme at 50 °C. (TIF) [file pone.0192488.s002.tif]

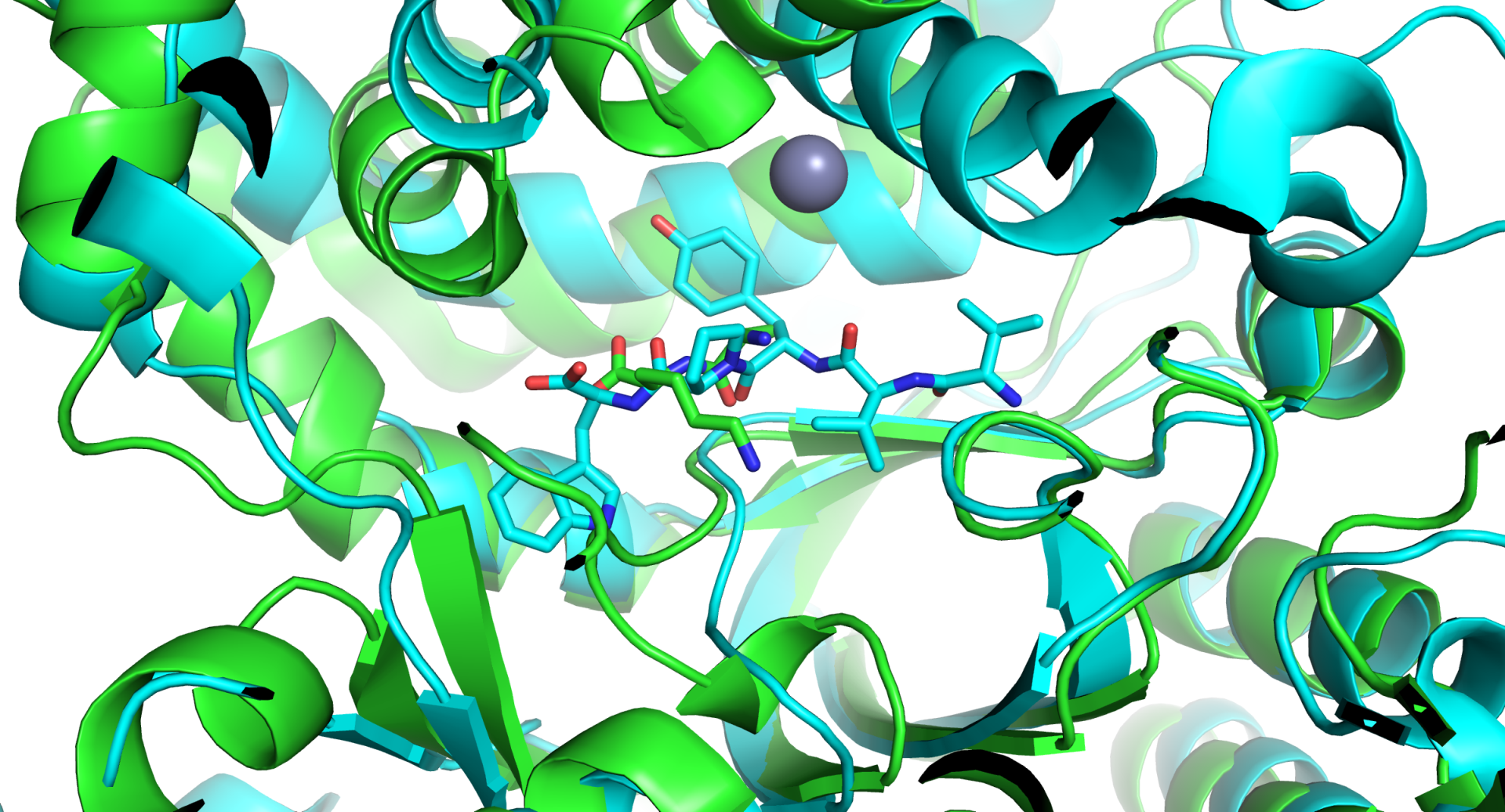

Supplement: S3 Fig — Lys-Ala dipeptide is shown with green sticks; tynorphin is shown with cyan sticks. Zinc ion is represented by a grey sphere. (TIF) [file pone.0192488.s003.tif]

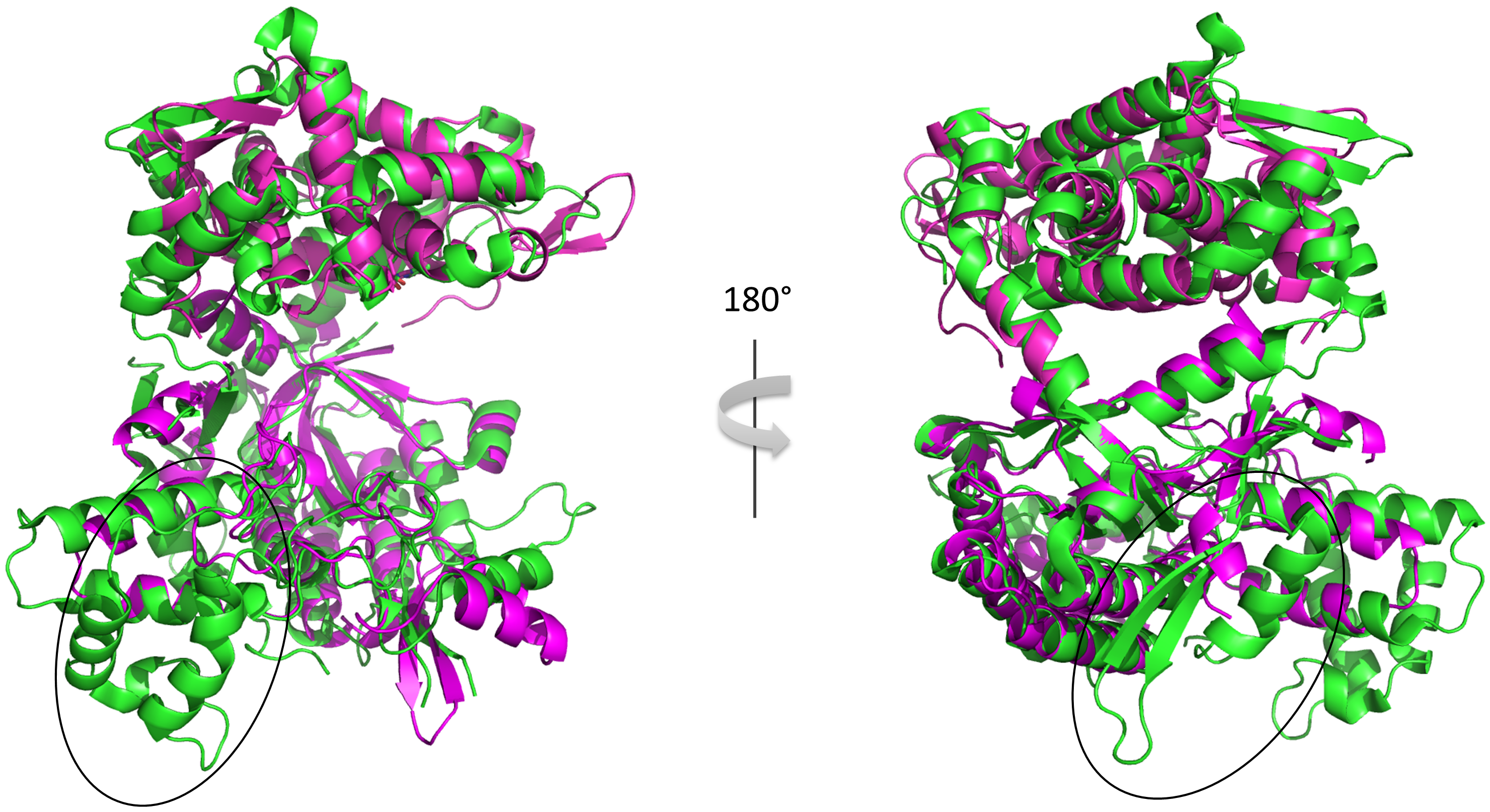

Supplement: S4 Fig — CaDPP III lacks α-helix-loop-α-helix motif (left) and two β-strand and α-helix (right). Missing motifs are marked with black ellipses. (TIF) [file pone.0192488.s004.tif]

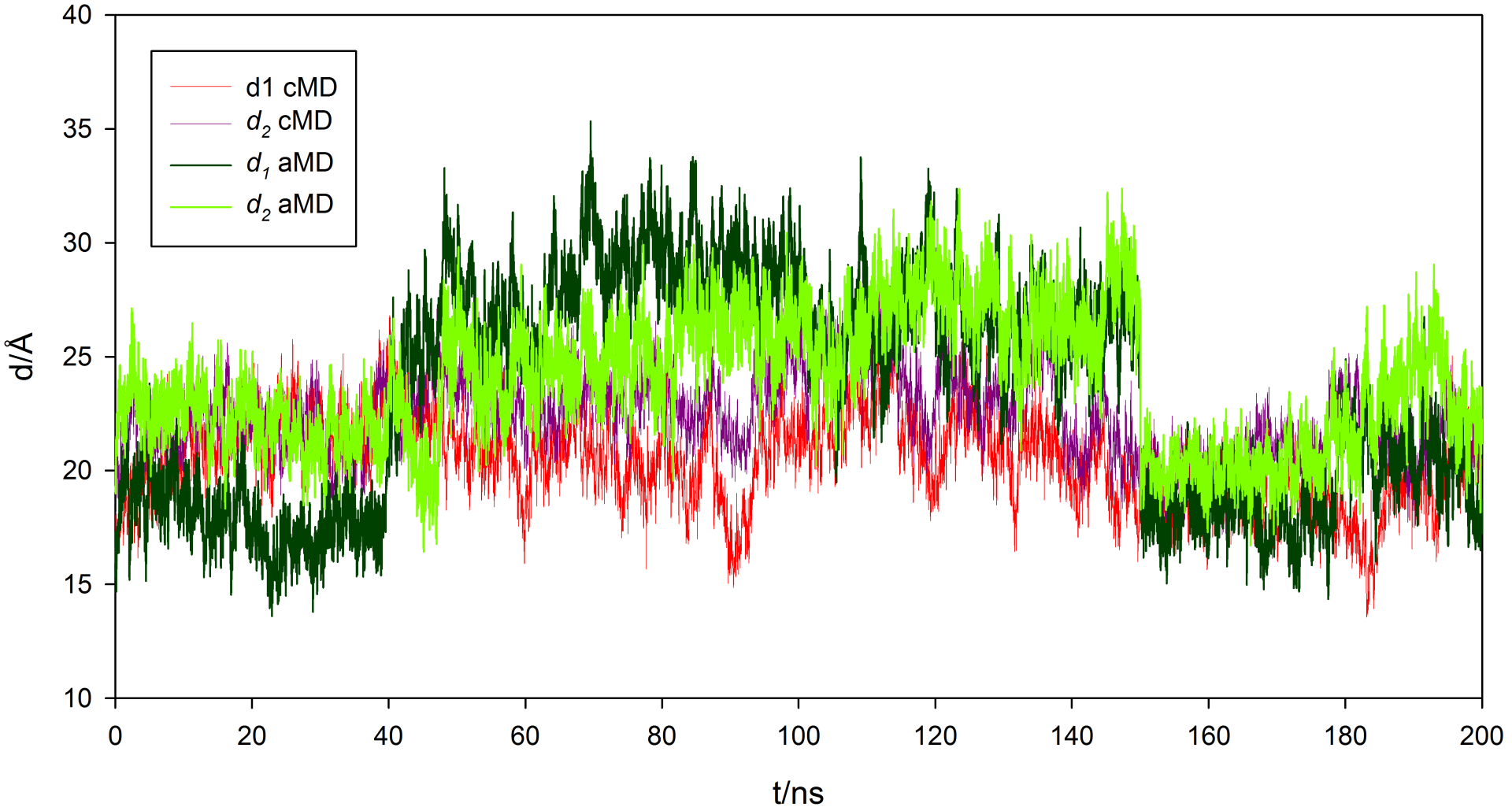

Supplement: S5 Fig — (TIF) [file pone.0192488.s005.tif]

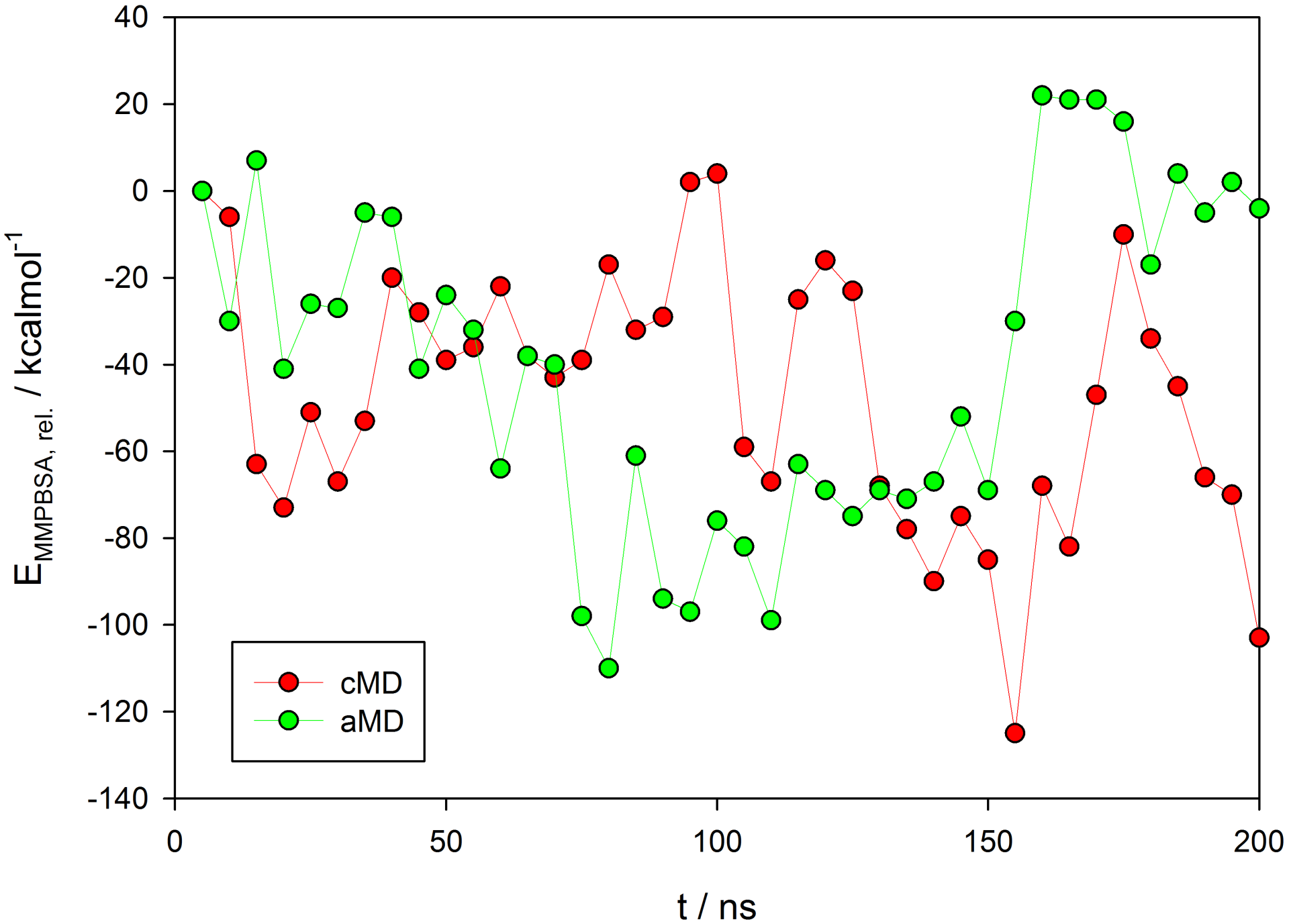

Supplement: S6 Fig — The values determined for the sets of structures sampled during 5 ns intervals in raw are shown. (TIF) [file pone.0192488.s006.tif]

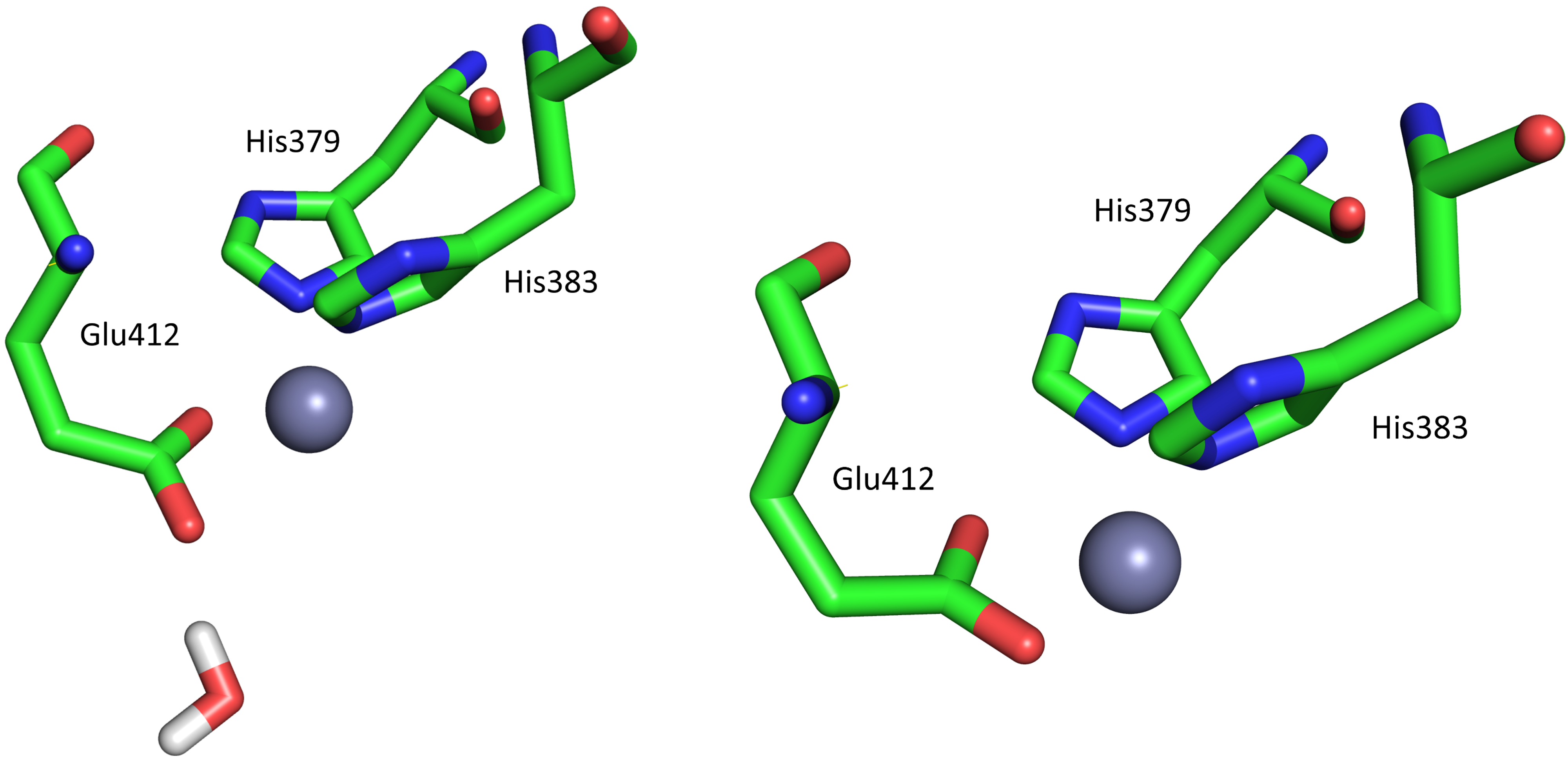

Supplement: S7 Fig — Zinc ion is shown as a grey sphere. (TIF) [file pone.0192488.s007.tif]

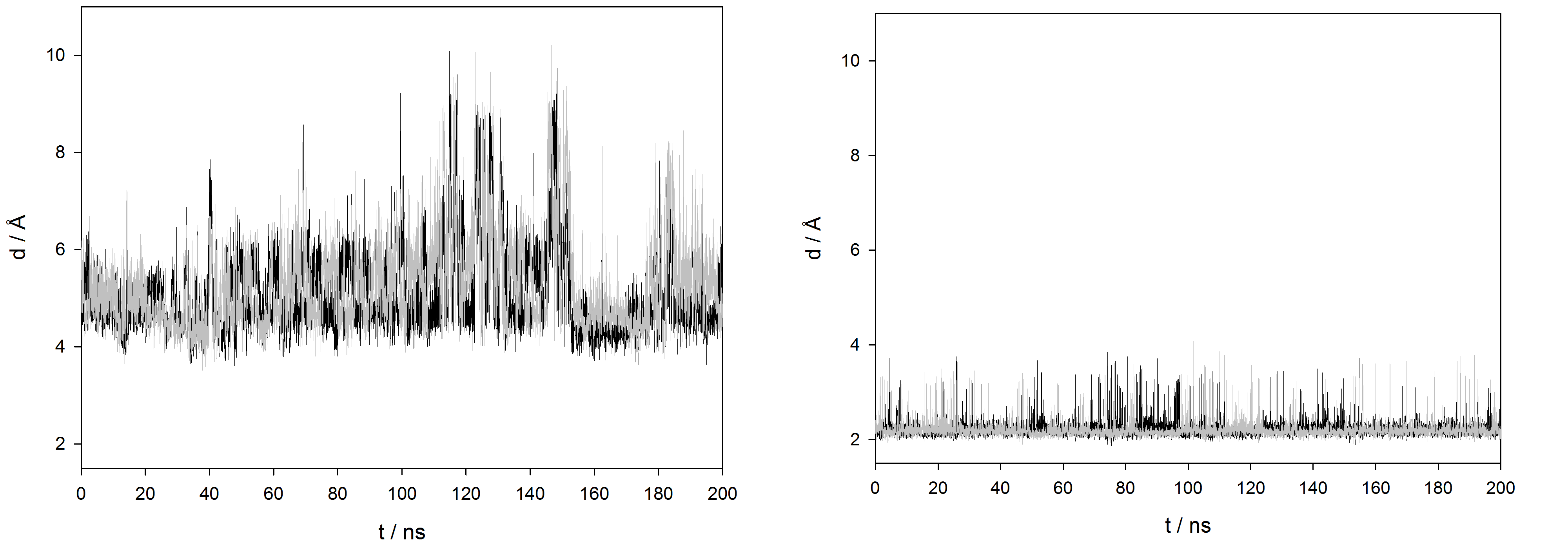

Supplement: S8 Fig — (TIF) [file pone.0192488.s008.tif]

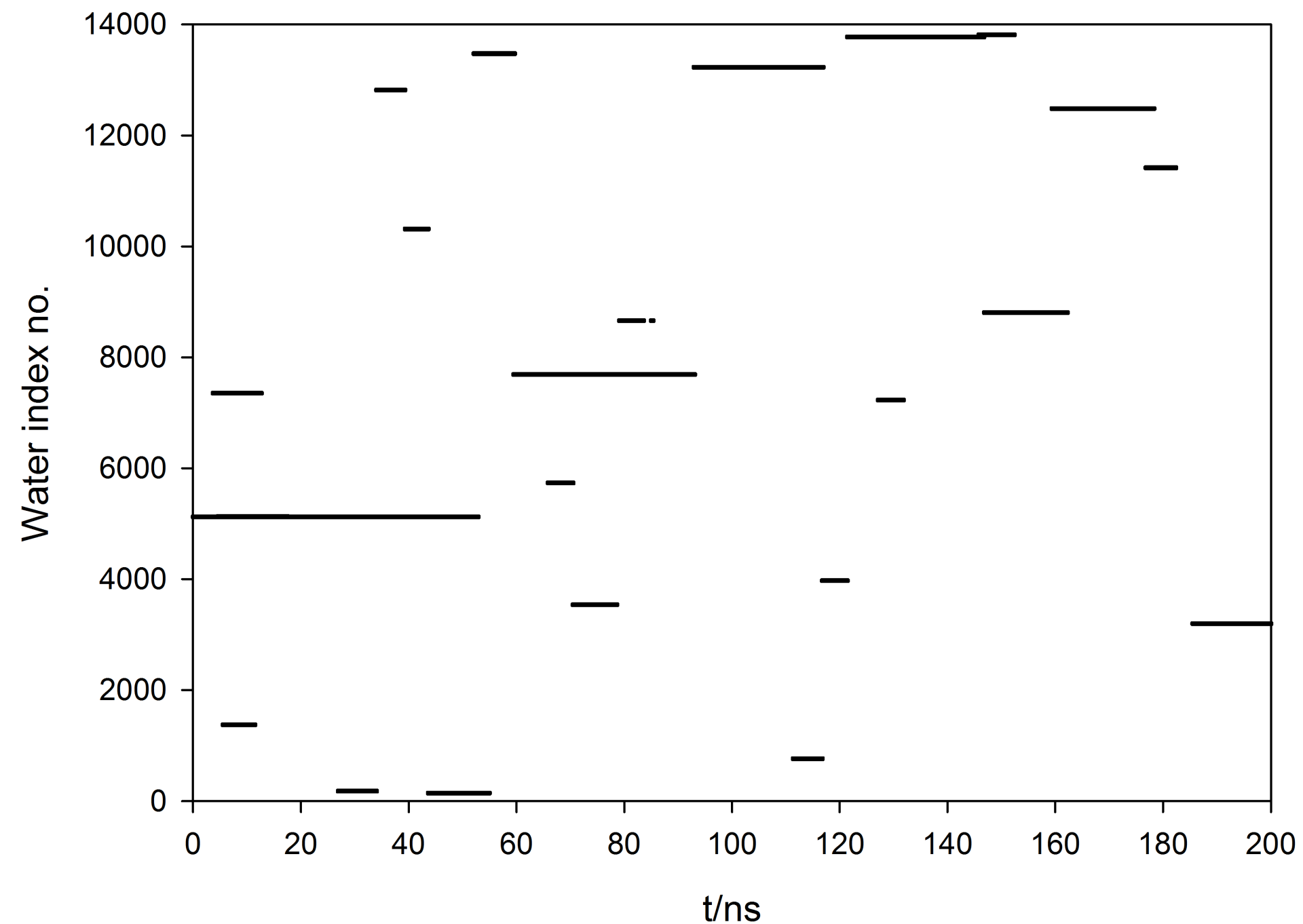

Supplement: S9 Fig — Water molecules appearing in less than 200 sampled frames are omitted. (TIF) [file pone.0192488.s009.tif]

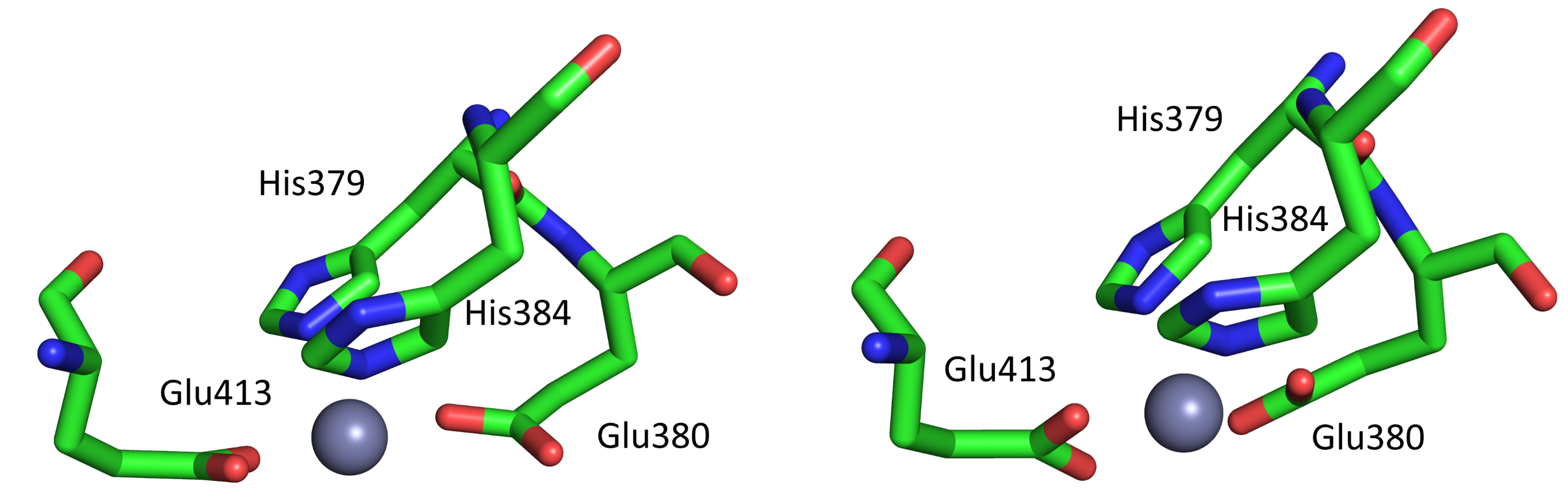

Supplement: S10 Fig — Zinc ion is shown as a grey sphere. (TIF) [file pone.0192488.s010.tif]

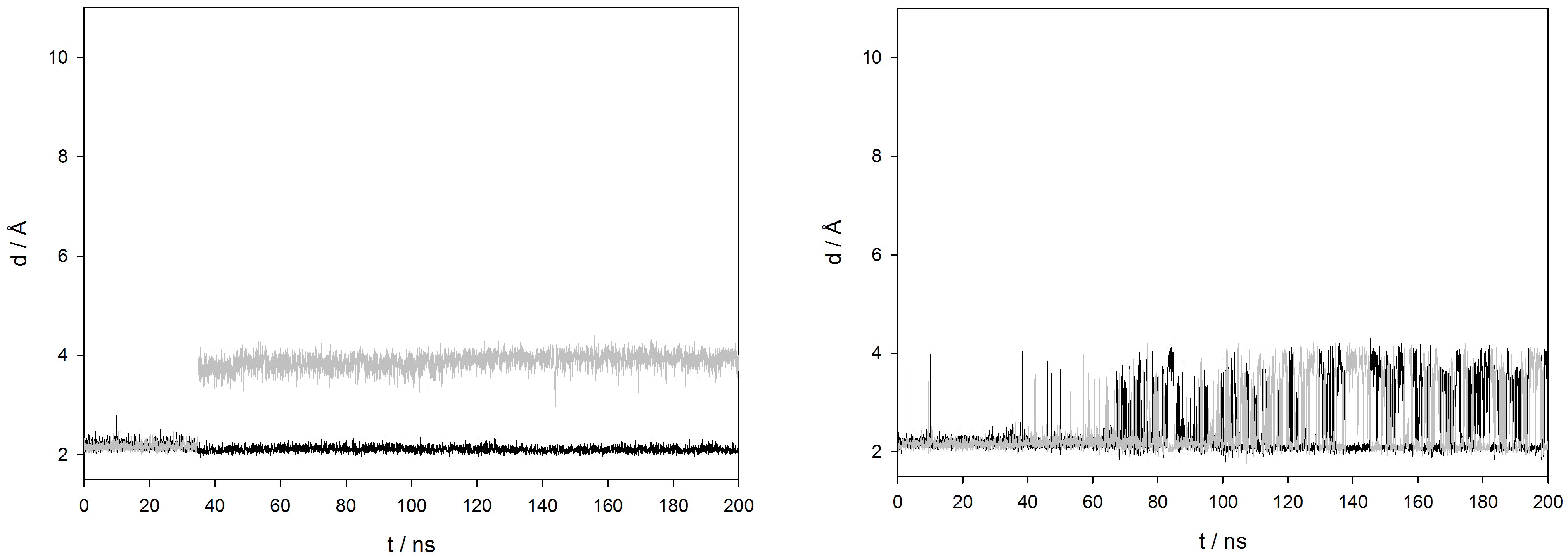

Supplement: S11 Fig — (TIF) [file pone.0192488.s011.tif]

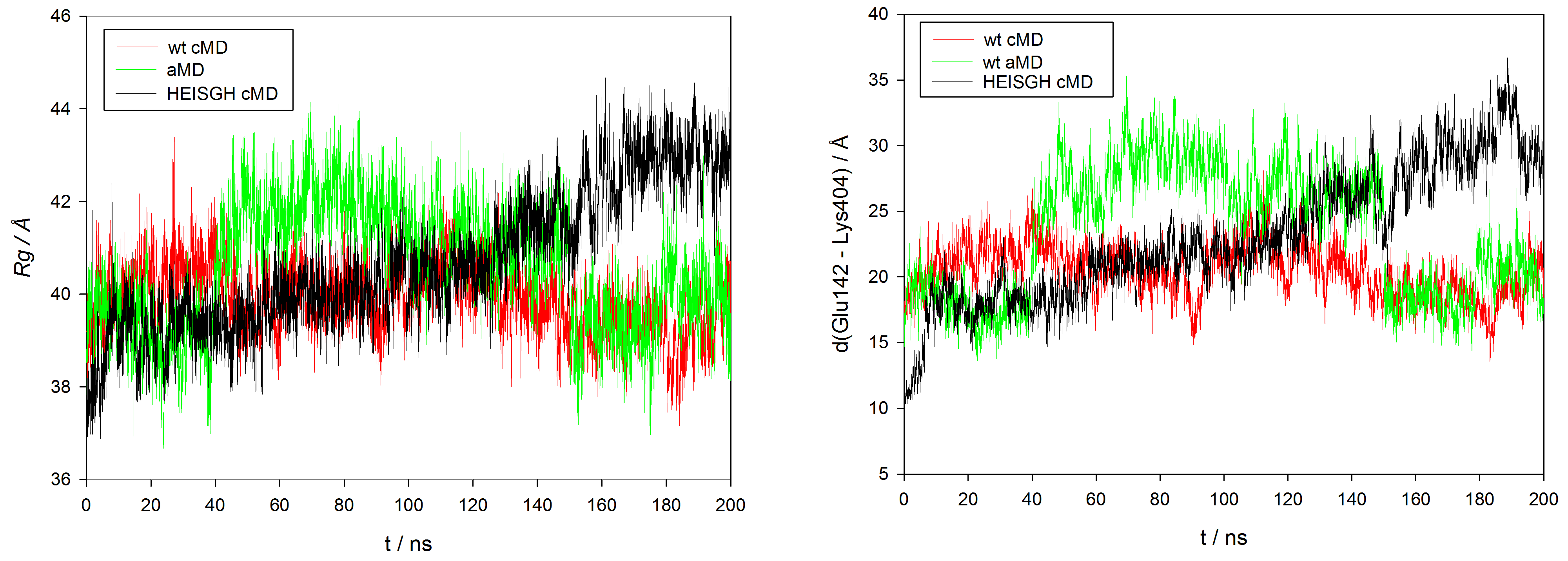

Supplement: S12 Fig — (TIF) [file pone.0192488.s012.tif]

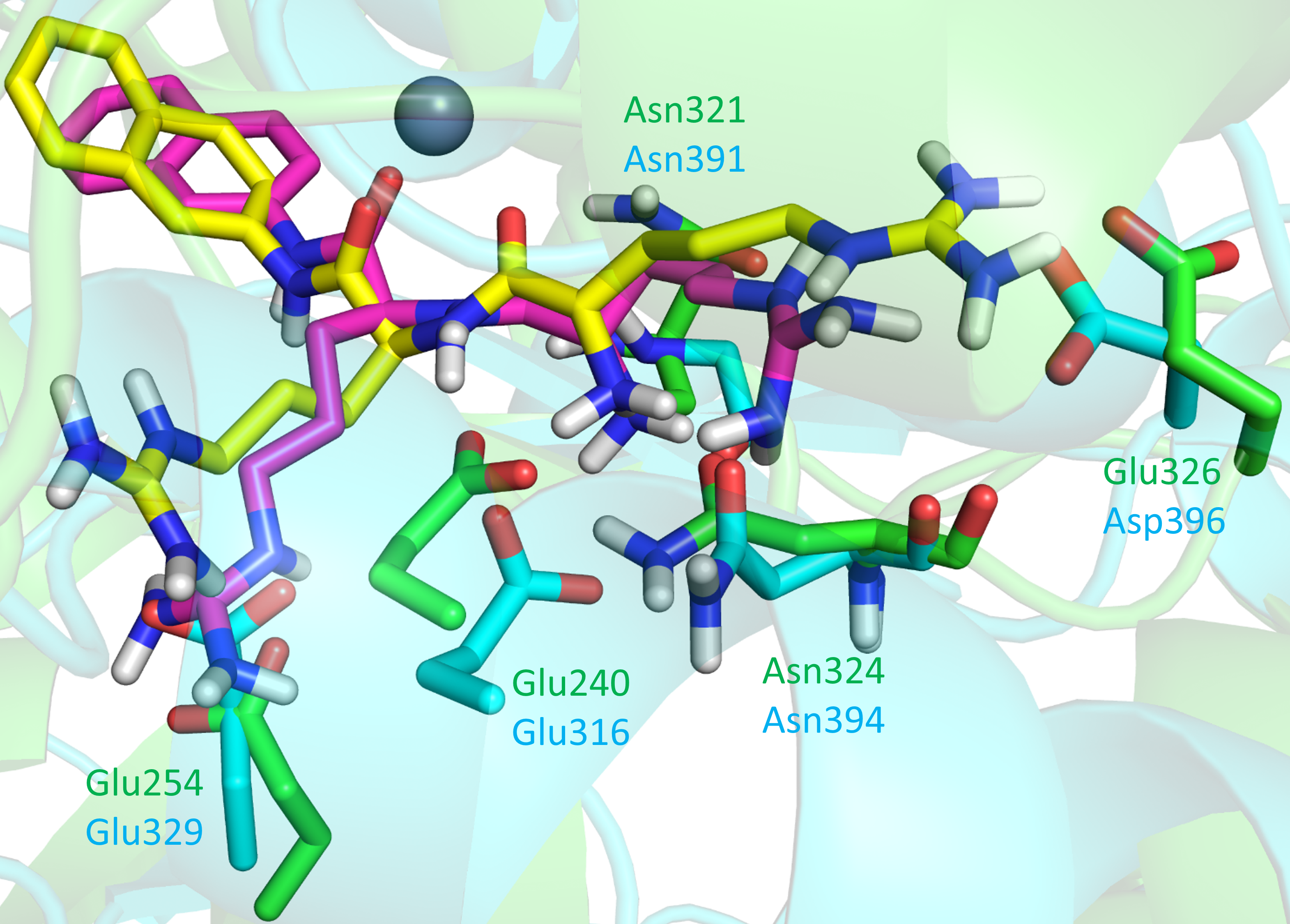

Supplement: S13 Fig — Zn2+ is shown as a grey sphere. (TIF) [file pone.0192488.s013.tif]
