# Supplementary material for: The first dipeptidyl peptidase III from a thermophile: Structural basis for thermal stability and reduced activity
Source: PLoS One. 2018 Feb 8;13(2):e0192488. doi: 10.1371/journal.pone.0192488 (PMC5805324; doi:10.1371/journal.pone.0192488)
Supplement: S1 Table — All values are given in kcal mol-1. (DOCX) [file pone.0192488.s014.docx]

**S1 Table.** The average energy values determined during the first 50 ns of MD simulation of the ligand-free enzyme using ff14SB and the parameters required for aMD simulations. All values are given in kcal mol^-1^.

| $\bar{E_{pot}}$^a^ | -159315 |
| --- | --- |
| $\bar{E_{dih}}$^a^ | 7027 |
| $E_{r}$ ^b^ | 1.0 |
| $E_{a}$^b^ | 0.1 |
| $E_{threshP}$ | -153955 |
| $\alpha_{P}$ | 5360 |
| $E_{threshD}$ | 7567 |
| $\alpha_{D}$ | 108 |

^a^Average potential and dihedral energies. ^b^*E*_r_ and *E*_a_ are the energy per residue and atom, respectively, used to calculate threshold energies and α.
