# Supplementary material for: The first dipeptidyl peptidase III from a thermophile: Structural basis for thermal stability and reduced activity
Source: PLoS One. 2018 Feb 8;13(2):e0192488. doi: 10.1371/journal.pone.0192488 (PMC5805324; doi:10.1371/journal.pone.0192488)
Supplement: S2 Table — All values are given in kcal mol-1. Er and Ea values are 1.0 and 0.1 kcal mol-1, respectively. (DOCX) [file pone.0192488.s015.docx]

**S2 Table.** The average energy values determined during the 50 ns of MD simulations of *Ca*DPP III – RRNA complex using ff14SB and the parameters required for aMD simulations. All values are given in kcal mol^-1^. *E*_r_ and *E*_a_ values are 1.0 and 0.1 kcal mol^-1^, respectively.

|  | ***Ca*DPP III – RRNA** | ***Ca*DPP III – GRNA** | ***Ca*DPPIII – GPNA** | ***Ca*DPPIII – GFNA** |
| --- | --- | --- | --- | --- |
| $\bar{E_{pot}}$^a^ | -199640 | -199500 | -212451 | -212296 |
| $\bar{E_{dih}}$^a^ | 7090 | 7071 | 7085 | 7078 |
| $E_{threshP}$ | -192988 | -192855 | -205407 | -205257 |
| $\alpha_{P}$ | 6651 | 6644 | 7044 | 7039 |
| $E_{threshD}$ | 7631 | 7614 | 7625 | 7618 |
| $\alpha_{D}$ | 108 | 108 | 108 | 108 |

^a^Average potential and dihedral energies.
