# Supplementary material for: The first dipeptidyl peptidase III from a thermophile: Structural basis for thermal stability and reduced activity
Source: PLoS One. 2018 Feb 8;13(2):e0192488. doi: 10.1371/journal.pone.0192488 (PMC5805324; doi:10.1371/journal.pone.0192488)
Supplement: S3 Table — (DOCX) [file pone.0192488.s016.docx]

**S3 Table.** Relative activity of *Ca*DPP III at pH 6-7 in 50 mM phosphate buffer, and pH 7-8.6 in 50 mM Tris HCl buffer at 37 and 50 °C.

| **Buffer** | **pH** | **Relative activity at 37°C / %** | **Relative activity at 50°C / %** |
| --- | --- | --- | --- |
| Na-PO_4_ | 6.0 | 13 | 6 |
|  | 6.2 | 17 | 8 |
|  | 6.7 | 24 | 16 |
|  | 7.0 | 58 | 20 |
| Tris-HCl | 7.0 | 100 | 100 |
|  | 7.4 | 98 | 79 |
|  | 8.0 | 69 | 75 |
|  | 8.6 | 59 | 60 |
