# Supplementary material for: The first dipeptidyl peptidase III from a thermophile: Structural basis for thermal stability and reduced activity
Source: PLoS One. 2018 Feb 8;13(2):e0192488. doi: 10.1371/journal.pone.0192488 (PMC5805324; doi:10.1371/journal.pone.0192488)
Supplement: S4 Table — (DOCX) [file pone.0192488.s017.docx]

**S4 Table.** The influence of effectors on *Ca*DPP III activity towards Arg_2_-2NA and Gly-Arg-2NA substrates.

| Effector | c(effector) / mM | RA Arg_2_-2NA/ % | RA Gly-Arg-2NA/ % |
| --- | --- | --- | --- |
| - | - | 100 | 100 |
| DTDP | 0.1 | 78.1 ± 6.5 | 43.2 ± 1.8 |
| pHMB | 0.001 | 94.4 ± 9.5 | 87.7 ± 7.8 |
| EDTA | 10 | 1.4 ± 2 | 0 |
| o-phenantrolin | 1 | 1.6 ± 0.1 | 0 |
| IAM | 10 | 85.3 ± 8.7 | 27.3 ± 15.4 |
| DTT | 0.035 | 100.4 ± 9.7 | 93.8 ± 2.1 |
| GSH | 0.035 | 86.4 ± 2 | 151 ± 8.5 |

RA - Relative activity towards Arg_2_-2NA substrate in comparison to the specific activity without the addition of effectors; relative activity is the average of two independent measurements.

DTDP- 4,4'-Dithiodipyridine; pHMB – 4-Hydroxymercuribenzoate; EDTA – ethylenediaminetetraacetic acid; IAM – Iodoacetamide; DTT – Dithiothreitol; GSH – reduced glutathione
