# Supplementary material for: The first dipeptidyl peptidase III from a thermophile: Structural basis for thermal stability and reduced activity
Source: PLoS One. 2018 Feb 8;13(2):e0192488. doi: 10.1371/journal.pone.0192488 (PMC5805324; doi:10.1371/journal.pone.0192488)
Supplement: S5 Table — (DOCX) [file pone.0192488.s018.docx]

**S5 Table.** Influence of metal ions on *Ca*DPP III peptidase activity towards Arg_2_-2-NA substrate measured at 50°C and pH 7.0.

| Metal ion | c(M^2+^) / µM | RA / % |
| --- | --- | --- |
| No metal | / | 100* |
| Co^2+^(CoCl_2_) | 5 | 104.6 ± 4.2 |
|  | 10 | 117.6 ± 6.7 |
|  | 50 | 123.1 ± 3.2 |
|  | 100 | 127.3 ± 6.2 |
| Mn^2+^(MnCl_2_) | 10 | 106.6 ±4.8 |
|  | 100 | 106.9 ± 6.8 |
| Mg^2+^(MgAc_2_) | 10 | 103.1 ± 1.4 |
|  | 100 | 104.3 ± 3.8 |
| Zn^2+^(Zn(OAc)_2_) | 2 | 97.5 ± 1 |
|  | 10 | 105.1 ± 2.7 |

RA - Relative activity towards Arg2-2NA substrate in comparison to the specific activity without the addition of metal ions which was 100.7 nmol min^-1^ mg^-1^; relative activity is the average of two independent measurements
