# Supplementary material for: The first dipeptidyl peptidase III from a thermophile: Structural basis for thermal stability and reduced activity
Source: PLoS One. 2018 Feb 8;13(2):e0192488. doi: 10.1371/journal.pone.0192488 (PMC5805324; doi:10.1371/journal.pone.0192488)
Supplement: S7 Table — The radius of gyration (Rg) was calculated for the protein backbone atoms. The distances d1 and d2 were calculated for Cα atoms. RMSD calculated for lower (RMSDLD) and upper domain (RMSDUD) with respect to the experimentally determined structure is given in the last two rows. (DOCX) [file pone.0192488.s020.docx]

**S7 Table.** Values of the geometric parameters used to describe degree and type of *Ca*DPP III closure determined in the most distinct enzyme structures, experimental and those obtained using accelerated MD simulations. The radius of gyration (R_g_) was calculated for the protein backbone atoms. The distances *d*_1_ and *d*_2_ were calculated for Cα atoms. RMSD calculated for lower (*RMSD*_LD_) and upper domain (*RMSD*_UD_) with respect to the experimentally determined structure is given in the last two rows.

| **geometric parameters** | **WT_E_** | **WT^c^_AMD_** | **WT^o^_AMD_** | **WT^C^_EQ_** |
| --- | --- | --- | --- | --- |
| *R*_g_ / Å | 23.5 | 23.2 | 24.5 | 23.0 |
| *d*_1_(E142-K404) / Å | 22.6 | 17.9 | 30.9 | 15.4 |
| *d*_2_(E330- K404) / Å | 23.2 | 22.1 | 30.6 | 19.4 |
| *RMSD*_LD_ / Å | 0 | 0.811 | 1.083 | 0.851 |
| *RMSD*_UD_ / Å | 0 | 0.793 | 0.713 | 0.660 |
