# Supplementary material for: The first dipeptidyl peptidase III from a thermophile: Structural basis for thermal stability and reduced activity
Source: PLoS One. 2018 Feb 8;13(2):e0192488. doi: 10.1371/journal.pone.0192488 (PMC5805324; doi:10.1371/journal.pone.0192488)
Supplement: S8 Table — Values of representative, the lowest energy structures* are given. (DOCX) [file pone.0192488.s021.docx]

**S8 Table.** Geometric parameters determined for the *Ca*DPP III complexes with Arg_2_-2-NA, Gly-Ala-2-NA, Gly-Phe-2-NA and Gly-Pro-2-NA during 150 ns of MD simulations. Values of representative, the lowest energy structures* are given.

| **geometric parameters** | ***Ca*DPP III- Arg_2_-2-NA** | ***Ca*DPP III- Gly-Arg-2-NA** | ***Ca*DPP III-Gly-Phe-2-NA** | ***Ca*DPP III-Gly-Pro-2-NA** |
| --- | --- | --- | --- | --- |
| *R*_g_^**^ / Å | 22.4 | 22.5 | 22.7 | 23.2 |
| *d*_1_(E142-K404) / Å | 10.8 | 10.8 | 12.1 | 15.5 |
| *d*_2_(E330- K404) / Å | 20.7 | 18.5 | 20.1 | 19.1 |
| *RMSD_LD_* / Å | 0.851 | 0.971 | 1.319 | 0.847 |
| *RMSD_UD_* / Å | 1.070 | 0.883 | 0.794 | 0.891 |

*structures from the interval in which the lowest MM-PBSA energies were calculated

**Amino acid residues 19 – 32 are omitted from R_g_ calculations due to the lack of secondary structure
