# Supplementary material for: The first dipeptidyl peptidase III from a thermophile: Structural basis for thermal stability and reduced activity
Source: PLoS One. 2018 Feb 8;13(2):e0192488. doi: 10.1371/journal.pone.0192488 (PMC5805324; doi:10.1371/journal.pone.0192488)
Supplement: S9 Table — The non-conserved amino acid residues are given in bold. (DOCX) [file pone.0192488.s022.docx]

**S9 Table.** Amino acid residues composition of the S1 and S2 subsites in the *Ca*, *Bt*, yeast and human DPPIII. The non-conserved amino acid residues are given in bold.

| **Subsites** | ***Ca*DPP III** | ***Bt*DPP III** | **Yeast DPP III** | **Human DPP III** |
| --- | --- | --- | --- | --- |
| S1 | **V235** | Y309 | Y327 | Y318 |
|  | E254 | E320 | E338 | E329 |
|  | **S307** | **L372** | F390 | F381 |
|  | **T317** | **A381** | P396 | P387 |
|  | G309 | G383 | G398 | G389 |
|  | **F320** | I384 | I397 | I390 |
|  | H379 | H448 | H460 | H450 |
|  | E412 | E476 | E517 | E508 |
|  | H460 | H533 | H578 | H568 |
| S2 | E240 | E307 | E325 | E316 |
|  | F320 | I384 | I399 | I390 |
|  | N321 | N385 | N400 | N391 |
|  | N324 | N388 | N403 | N394 |
|  | **E326** | **N390** | D405 | D396 |
|  | R329 | R393 | R408 | R399 |
|  | H383 | H453 | H465 | H455 |
|  | **V399** | **P464** | W504 | W495 |
|  | **K400** | D465 | **G505** | D496 |
|  | S408 | S472 | **G513** | S504 |
|  | E411 | E475 | E516 | E507 |
|  | E412 | E476 | E517 | E508 |
