# Supplementary material for: The first dipeptidyl peptidase III from a thermophile: Structural basis for thermal stability and reduced activity
Source: PLoS One. 2018 Feb 8;13(2):e0192488. doi: 10.1371/journal.pone.0192488 (PMC5805324; doi:10.1371/journal.pone.0192488)
Supplement: S10 Table — The analysis was performed for the lowest-energy 5 ns long fragments of the 150 ns long (100 ns cMD + 50 ns aMD) trajectories used to calculate the MM-PBSA energies. The hydrogen bonds occurring <5% in all of the sampled structures are omitted. (DOCX) [file pone.0192488.s023.docx]

**S10 Table.** Number of charged amino acid residues within 6 Å of tynorphin bound into the enzyme active site

|  | **h.DPP III** | ***Bt*DPP III** | ***Ca*DPP III** |
| --- | --- | --- | --- |
| Glu | 6 | 4 | 7 |
| Asp | 1 | 2 | 2 |
| ∑ | 7 | 6 | 9 |
| Arg | 4 | 4 | 3 |
| Lys | 3 | 1 | 3 |
| ∑ | 7 | 5 | 6 |
