# Supplementary material for: The first dipeptidyl peptidase III from a thermophile: Structural basis for thermal stability and reduced activity
Source: PLoS One. 2018 Feb 8;13(2):e0192488. doi: 10.1371/journal.pone.0192488 (PMC5805324; doi:10.1371/journal.pone.0192488)
Supplement: S11 Table — (DOCX) [file pone.0192488.s024.docx]

**S11 Table.** Hydrogen bonds population (%) for the HEISGH mutant complex with Arg_2_-2NA. The analysis was performed for the lowest-energy 5 ns long fragments of the 150 ns long (100 ns cMD + 50 ns aMD) trajectories used to calculate the MM-PBSA energies. The hydrogen bonds occurring <5% in all of the sampled structures are omitted.

| **Acceptor** | **Arg_2_-2NA** |
| --- | --- |
| Glu240 | 117.2 |
| Tyr242 | 17.2 |
| Glu254 | 73.6 |
| Asp310 | 159.6 |
| Val315 | 94.8 |
| Thr317 | 51.6 |
| Ala319 | 52.4 |
| Asn321 | 98.4 |
| Asn324 | 117.2 |
| Glu399 | 157.2 |
| Tyr408 | 6.8 |
| Glu413 | 7.2 |
| **Donor** | **Arg_2_-2NA** |
| Glu240 | 16.0 |
| Phe320 | 10.4 |
| Asn321 | 64.4 |
